# Methylation patterns in serum DNA for early identification of disseminated breast cancer

**DOI:** 10.1186/s13073-017-0499-9

**Published:** 2017-12-22

**Authors:** Martin Widschwendter, Iona Evans, Allison Jones, Shohreh Ghazali, Daniel Reisel, Andy Ryan, Aleksandra Gentry-Maharaj, Michal Zikan, David Cibula, Johannes Eichner, Marianna Alunni-Fabbroni, Julian Koch, Wolfgang J. Janni, Tobias Paprotka, Timo Wittenberger, Usha Menon, Benjamin Wahl, Brigitte Rack, Harri Lempiäinen

**Affiliations:** 1 Department of Women’s Cancer, UCL Elizabeth Garrett Anderson Institute for Women’s Health, University College London, Medical School Building, 74 Huntley Street, London, WC1E 6AU UK; 2 Gynaecologic Oncology Center, Department of Obstetrics & Gynaecology, First Faculty of Medicine & General University Hospital, Charles University Prague, Prague, Czech Republic; 3 Genedata AG, Margarethenstrasse 38, 4053 Basel, Switzerland; 4 Department of Gynaecology and Obstetrics, Klinikum Innenstadt, Ludwig-Maximilians Universitaet Muenchen, Maistrasse 11, 80337 Munich, Germany; 5 Department of Gynaecology and Obstetrics, University Hospital Ulm, Prittwitzstrasse 43, 89075 Ulm, Germany; 6 GATC Biotech AG, Jakob-Stadler-Platz 7, 78467 Konstanz, Germany; 7 Boehringer Ingelheim Pharma, GmbH & Co. KG, Target Discovery Research, Biberach, Germany

**Keywords:** Cell-free DNA, DNA methylation, Serum DNA, Breast cancer, Early diagnosis, Personalized treatment

## Abstract

**Background:**

Monitoring treatment and early detection of fatal breast cancer (BC) remains a major unmet need. Aberrant circulating DNA methylation (DNAme) patterns are likely to provide a highly specific cancer signal. We hypothesized that cell-free DNAme markers could indicate disseminated breast cancer, even in the presence of substantial quantities of background DNA.

**Methods:**

We used reduced representation bisulfite sequencing (RRBS) of 31 tissues and established serum assays based on ultra-high coverage bisulfite sequencing in two independent prospective serum sets (n = 110). The clinical use of one specific region, EFC#93, was validated in 419 patients (in both pre- and post-adjuvant chemotherapy samples) from SUCCESS (Simultaneous Study of Gemcitabine-Docetaxel Combination adjuvant treatment, as well as Extended Bisphosphonate and Surveillance-Trial) and 925 women (pre-diagnosis) from the UKCTOCS (UK Collaborative Trial of Ovarian Cancer Screening) population cohort, with overall survival and occurrence of incident breast cancer (which will or will not lead to death), respectively, as primary endpoints.

**Results:**

A total of 18 BC specific DNAme patterns were discovered in tissue, of which the top six were further tested in serum. The best candidate, EFC#93, was validated for clinical use. EFC#93 was an independent poor prognostic marker in pre-chemotherapy samples (hazard ratio [HR] for death = 7.689) and superior to circulating tumor cells (CTCs) (HR for death = 5.681). More than 70% of patients with both CTCs and EFC#93 serum DNAme positivity in their pre-chemotherapy samples relapsed within five years. EFC#93-positive disseminated disease in post-chemotherapy samples seems to respond to anti-hormonal treatment. The presence of EFC#93 serum DNAme identified 42.9% and 25% of women who were diagnosed with a fatal BC within 3–6 and 6–12 months of sample donation, respectively, with a specificity of 88%. The sensitivity with respect to detecting fatal BC was ~ 4-fold higher compared to non-fatal BC.

**Conclusions:**

Detection of EFC#93 serum DNAme patterns offers a new tool for early diagnosis and management of disseminated breast cancers. Clinical trials are required to assess whether EFC#93-positive women in the absence of radiological detectable breast cancers will benefit from anti-hormonal treatment before the breast lesions become clinically apparent.

**Electronic supplementary material:**

The online version of this article (doi:10.1186/s13073-017-0499-9) contains supplementary material, which is available to authorized users.

## Background

Breast cancer (BC) is by far the most frequently occurring cancer in women. Every year 522,000 women die from BC [1].

Mammography is used as a screening tool for early diagnosis but has its limitations due to over-diagnosis and a modest impact on mortality [2]. Recent evidence demonstrates that dissemination might occur during the very early stages of tumor evolution and before clinical manifestation of the cancer in the breast [3]. The analyses of circulating markers in order to identify women with disseminated disease before diagnosis have not been successful [4].

Numerous studies have demonstrated that patients with disseminated tumor cells in the bone marrow [5–7] or circulating tumor cells (CTCs) [8–12] have an inferior prognosis. The immunocytochemical detection of CTCs is reliant upon the isolation of intact cells.

Adjuvant systemic treatment has reduced BC mortality over the last two to three decades [13]. The current strategy guiding administration of adjuvant systemic treatment is reliant upon primary tumor characteristics. However, systemic relapse and subsequent death are caused by disseminated disease whose biological properties may be very different to those comprising the primary tumor [14].

Recently, markers based on DNA shed from tumor cells have shown great promise in monitoring treatment response and predicting prognosis [15–19]. However, efforts to characterize the cancer genome have shown that only a few genes are frequently mutated in cancer and the site of mutation per gene differs across tumors [20]. A further limitation is that current technology only allows for the detection of a mutant allele fraction of 0.1% [15, 21].

Over the last decade, DNA methylation (DNAme) has been shown to be a hallmark of cancer [22] and occurs very early in BC development [23]. DNAme is centered around specific regions (CpG islands) [22] and is chemically and biologically stable. This enables the development of early detection tools and personalized treatment, based upon the analysis of cell-free DNA contained within serum or plasma [24–29]. However, two major challenges have to be overcome: (1) the very low abundance of cancer-DNA in the blood; and (2) the high level of “background DNA” shed from white blood cells (WBC) [30] in banked samples.

To date, virtually all research work has been carried out in relatively small studies and focused on the analyses of cell-free DNAme in metastatic/relapsed breast cancers using markers from previously published studies [31]. In our study we: (1) used an epigenome-wide approach to identify new markers which indicate disseminated breast cancer; (2) analyzed the top marker in 419 primary non-metastatic patients before (i.e. immediately after resection of the primary breast cancer) and after adjuvant chemotherapy; and, most importantly (3) analyzed the marker in 925 healthy women who either remained healthy or developed fatal or non-fatal BC within the first three years after serum sample donation.

## Methods

### Patients and sample collection

**Fig. 1:**
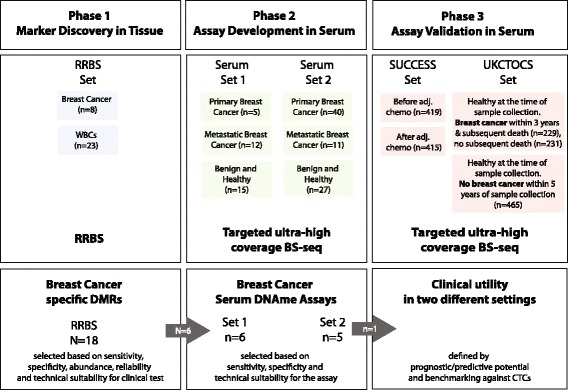
Study design. Using reduced representation bisulfite sequencing (RRBS), 31 human tissue samples were analyzed to identify a total of 18 regions which underwent thorough technical validation. Six regions were selected whose methylation status has been analyzed in two sets consisting of 110 serum samples. One marker (EFC#93) has been validated in two independent settings: (1) in SUCCESS study serum samples from BC patients before and after chemotherapy; and (2) in UKCTOCS serum samples from women before BC diagnosis (within three years) or who remained healthy for five years

We used a total of 31 tissues and 1869 serum samples (Fig. 1). In Phase 1, we analyzed breast cancer tissue and WBCs in order to identify breast cancer specific DNAme markers. In Phase 2, we established serum DNAme assays using serum sets 1 and 2, collected from women attending hospitals in London, Munich, and Prague where they were invited and consented. Blood samples (20–40 mL) were obtained (in VACUETTE® Z Serum Sep Clot Activator tubes), centrifuged at 3000 rpm for 10 min, and serum collected and stored at – 80 °C. Finally, Phase 3 was initiated to validate the top marker performance by using serum samples from two large clinical studies: (1) from 419 patients recruited within the SUCCESS trial [10] (ClinicalTrial.gov registration ID is NCT02181101), where bloods were taken before and after chemotherapy and (within 96 h) sent to the laboratory for CTC assessment and serum samples stored (Additional file 1: Figure S1); and (2) from UKCTOCS [32] (ClinicalTrial.gov registration ID is NCT00058032), where serum samples were used from: (i) 229 women diagnosed with BC within the first three years after serum sample donation and subsequently died during follow-up; (ii) 231 matched women who developed BC within three years after sample donation and were alive at the end of follow-up; and (iii) 465 women who did not develop BC within five years after sample donation (Additional file 1: Figure S2). Blood samples from all UKCTOCS volunteers were spun down for serum separation after having been transported at room temperature from trial centers to the central laboratory. The median time between sample collection and centrifugation was 22.1 h. Only 1 mL of serum per UKCTOCS volunteer was available. All patients provided written informed consent.

### Isolation and bisulfite modification of DNA

DNA was isolated from tissue and serum samples at GATC Biotech (Konstanz, Germany). Tissue DNA was quantified using NanoDrop™ and Qubit™, and the size was assessed by agarose gel electrophoresis. Serum DNA was quantified using the Agilent Fragment Analyzer and the High Sensitivity Large Fragment Analysis Kit (AATI, USA). DNA was bisulfite converted at GATC Biotech.

### DNAme analysis in tissue

Genome-wide methylation analysis was performed by reduced representation bisulfite sequencing (RRBS) at GATC Biotech. DNA was digested with MspI followed by size selection of the library, providing enhanced coverage for the CpG-rich regions [33, 34]. The digested DNA was adapter-ligated, bisulfite-modified, and polymerase chain reaction (PCR)-amplified. The libraries were sequenced on Illumina’s HiSeq 2500. Analysis of the first samples sequenced with a 100-bp paired-end mode showed that the library insert size was small. Therefore, the remaining samples were sequenced with a 50-bp paired-end mode. Using Genedata Expressionist® for Genomic Profiling v9.1, we established a bioinformatics pipeline for the detection of cancer specific differentially methylated regions (DMRs). The most promising DMRs were taken forward for the development and validation of serum-based clinical assays.

### Targeted ultra-high coverage bisulfite sequencing of serum DNA

Targeted bisulfite sequencing libraries were prepared at GATC Biotech. Bisulfite modification was performed with 1 mL serum equivalent. A two-step PCR approach was used to test up to three different markers per modified DNA sample. The first PCR amplifies the target region and adds linker sequences which are used in the second PCR to add barcodes for multiplexing and sequences needed for sequencing. Ultra-high coverage sequencing was performed on Illumina’s MiSeq or HiSeq 2500 with a 75-bp or 125-bp paired-end mode, respectively.

### Data analyses

**Fig. 2:**
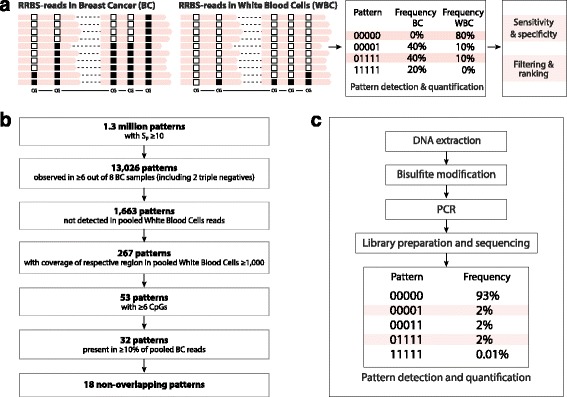
Principles of methylation pattern discovery in tissue (**a**, **b**) and analyses in serum (**c**). **a** RRBS was used in tissue samples in order to identify CpG methylation patterns that are able to discriminate breast cancer from white blood cells (which were deemed to be the most abundant source of cell-free DNA). “0” represents an unmethylated CpG and “1” represents a methylated CpG. An example of region EFC#93 is provided which is a 136-bp-long region containing five linked CpGs. The cancer pattern consists of reads in which all linked CpGs are methylated, indicated by “11111.” **b** RRBS data have been processed through a bioinformatic pipeline to identify the most promising markers. **c** The principles of the serum DNA methylation assay

Genedata Expressionist® for Genomic Profiling was used to map reads to human genome version hg19, identify regions with tumor-specific methylation patterns, quantify the occurrence of those patterns, and calculate relative pattern frequencies per sample. Pattern frequencies were calculated as number of reads containing the pattern divided by total reads covering the pattern region. Methylation patterns are represented in terms of a binary string, where the methylation state of each CpG site is denoted by “1” if methylated or “0” if unmethylated. The algorithm that we developed scans the whole genome and identifies regions that contain at least ten aligned paired-end reads. These read bundles are split into smaller regions of interest (ROIs) which contain at least 4 CpGs in a stretch of < 150 bp. For each region and tissue/sample, the absolute frequency (number of supporting reads) for all observed methylation patterns was determined (Fig. 2a). This led to the discovery of tens of millions of patterns per tissue/sample. The patterns were filtered in a multi-step procedure to identify the methylation patterns specifically occurring in tumor samples. To increase the sensitivity and specificity of our pattern discovery procedure, we pooled reads from different tumor or WBC samples and scored patterns based on over-representation within tumor tissue. The results were summarized in a specificity score, Sp, which reflects the cancer specificity of the patterns. After applying a cut-off of Sp ≥ 10, 1.3 million patterns for BC remained and were further filtered according to the various criteria detailed in Fig. 2b (further details are provided in Additional file 2).

The 95% confidence intervals (CI) for sensitivity and specificity have been calculated according to the efficient-score method [35]. The endpoints were defined according to the STEEP criteria, with relapse-free survival and overall survival as the primary endpoints. The product-limit method according to Kaplan–Meier was used to estimate survival. The survival estimates in different groups were compared using the log-rank test. The Cox proportional hazards regression model was used for the analyses taking into account all variables simultaneously.

Further details on samples and methods can be found in Additional file 2.

## Results

The samples, techniques, and purpose of the three phases used in this study (marker discovery, assay development, and assay validation) are summarized in Fig. 1. We first identified DMRs based on their methylation patterns and frequencies in relevant genomic regions, within a BC tissue panel. Methylation patterns with high specificity for breast cancer tissue were identified using the procedure described in Fig. 2b.

The selected 18 BC specific patterns identified by RRBS, were further validated using bisulfite sequencing. Thirty-one bisulfite sequencing primer pairs (1–3 per region) were designed and technically validated to determine PCR efficiency and sensitivity. A dilution series obtained by mixing fully unmethylated (i.e. whole genome amplified DNA) with fully methylated DNA (i.e. whole genome amplified DNA treated with CpG methyltransferase) was used to select six reactions which showed good coverage after sequencing (> 10^4^ reads) and sensitivity in highly diluted (<1:10^4^) samples (Additional file 3: Table S1). The best six reactions were taken into Phase 2, for further testing and assay development, in prospectively collected serum sets. We used ultra-deep bisulfite sequencing to develop assays for these candidate regions in 32 serum samples from Serum Set 1 (Figs. 1 and 2c). Five of the six reactions showed good sensitivity and specificity (particularly when discriminating between metastatic and primary BC), based on the abundance of tumor-specific patterns (see Additional file 1: Figure S3 for a complete overview of pattern counts from region EFC#93) and were selected for further validation in Serum Set 2 (*n* = 78). DNA methylation marker EFC#93, which was identified in RRBS as a region of ten linked CpGs methylated in BC, was optimized to a pattern of five linked CpGs and showed the best sensitivity and specificity independently in Set 1 and 2 (Additional file 1: Figure S4). A statistically higher pattern frequency, for the optimized marker EFC#93, was observed in the metastatic BC groups compared to the healthy/benign lesions or primary BC groups, in both Sets 1 and 2. This translates to an area under the curve (AUC) of a receiver operating characteristics (ROC) curve of 0.850 (95% CI = 0.745–0.955, *P* = 0.000004) and 0.845 (95% CI = 0.739–0.952, *P* = 0.000004) to discriminate healthy/benign lesions or primary BC from metastatic BC in Set 1 and Set 2, respectively. When Set 1 and 2 data were combined, the pattern frequency threshold was set to 0.0008 (i.e. 8 in 10,000 reads demonstrated methylation at all CpGs in the EFC#93 region), which led to a sensitivity of 60.9% and a specificity of 92.0% with respect to identifying metastatic BC (Additional file 1: Figure S4).

**Fig. 3:**
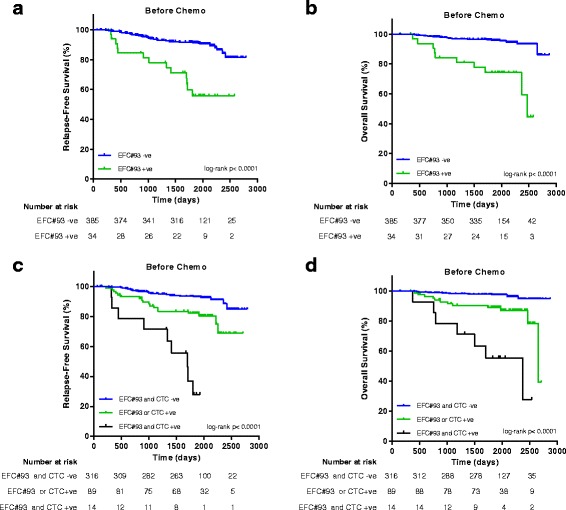
EFC#93 serum DNAme and CTC analyses in the SUCCESS trial in samples taken before chemotherapy. Kaplan–Meier analysis for relapse-free survival (**a**) and overall survival (**b**) according to the presence (EFC#93 pattern frequency ≥ 0.00008) or absence (EFC#93 pattern frequency < 0.00008) of marker EFC#93 before chemotherapy. Kaplan–Meier analysis for relapse-free survival (**c**) and overall survival (**d**) according to the presence/absence of EFC#93 and CTCs. *P* values from a two-sided log-rank test. *CTC–* no CTC present, *CTC+* at least one CTC present

EFC#93 was then validated for use as a prognostic and predictive BC marker in clinical trial samples (Fig. 1). As expected, due to delayed sample processing within these trials, serum samples from both SUCCESS and UKCTOCS contained high levels of contaminating WBC DNA, leading to dilution of the cancer signal (Additional file 1: Figure S5). In order to adjust for this, we made an a priori decision to reduce the threshold for EFC#93 pattern frequency by a factor of 10 to 0.00008 (i.e. 8/100,000 reads demonstrated methylation at all five linked CpGs within the EFC#93 region). Table 1 shows SUCCESS patient characteristics, correlated with EFC#93 positivity/negativity, before and after chemotherapy. Using our predetermined threshold, EFC#93 positivity was significantly associated with CTC presence, both before and after chemotherapy (Chi-square test, *P* < 0.01, Table 1) although ECF#93 pattern frequencies were not significantly different in samples from patients with either no, 1–4, or > 4 CTCs detected, respectively (Additional file 1: Figure S6). Patients who underwent breast-conserving therapy were more likely to be EFC#93-negative compared to patients who underwent a mastectomy; this is in all probability explained by the fact that patients which presented with larger tumors tended to be EFC#93-positive and would not have been eligible for breast-conserving surgery. This is consistent with the findings that EFC#93 positivity after chemotherapy is significantly (*P* = 0.014) less frequently observed in early stage (T1) compared to late stage (T2–4) cancers. None of the other clinical–pathological features correlated with cell-free DNA methylation of EFC#93 (Table 1). EFC#93 serum positivity before chemotherapy was a very strong marker of poor prognosis, for both relapse-free and overall survival (Table 2 and Fig. 3a and b). This was independent of the prognostic capability of CTCs (Additional file 1: Figures S7 and S8). Hazard ratios (HRs) (95% CI) for overall survival in the multivariable model were 5.973 (2.634–13.542) and 3.623 (1.681–7.812) for EFC#93 and CTCs, respectively (Table 2). Patients who were CTC-positive and EFC#93-positive had an extremely poor outcome, with > 70% of these patients relapsing within five years (Fig. 3c and d). Neither serum marker EFC#93 nor CTCs alone were predictive of the outcome in samples collected after chemotherapy (Additional file 1: Figures S9 and S10).

**Table 1:** SUCCESS patient characteristics before and after chemotherapy for EFC#93 serum DNAme

Characteristic		Before chemotherapy			After chemotherapy		
		EFC#93– (%)	EFC#93+ (%)	*P* value^a^	EFC#93– (%)	EFC#93+ (%)	*P* value^a^
Patients (n)		385 (91.9)	34 (8.1)		371 (89.4)	44 (10.6)	
Age (mean ± SD)		53.7 ± 10.3	55.2 ± 10.1	0.380	53.5 ± 10.4	56.2 ± 9.3	0.097
Menopausal status	Premenopausal	165 (42.9)	15 (44.1)	1.000	165 (44.5)	15 (34.1)	0.202
	Postmenopausal	220 (57.1)	19 (55.9)		206 (55.5)	29 (65.9)	
Stage (T)	T1	158 (41.0)	9 (26.5)	0.110	157 (42.3)	10 (22.7)	0.014
	T2–4	227 (59.0)	25 (73.5)		214 (57.7)	34 (77.3)	
Nodes (N)	NO	130 (33.9)	7 (20.6)	0.130	124 (33.4)	13 (30.2)	0.735
	N1–3	254 (66.1)	27 (79.4)		247 (66.6)	30 (69.8)	
Histology	Invasive ductal	310 (80.5)	25 (73.5)	0.370	296 (79.8)	36 (81.8)	0.844
	Others	75 (19.5)	9 (26.5)		75 (20.2)	8 (18.2)	
Grading	Grade 1/2	199 (51.7)	16 (47.1)	0.721	190 (51.2)	23 (52.3)	1.000
	Grade 3	186 (48.3)	18 (52.9)		181 (48.8)	21 (47.7)	
Estrogen (ER) receptor	ER–	128 (33.2)	10 (29.4)	0.708	128 (34.5)	10 (22.7)	0.130
	ER+	257 (66.8)	24 (70.6)		243 (65.5)	34 (77.3)	
Progesterone (PR) receptor	PR–	155 (40.4)	11 (32.4)	0.465	150 (40,5)	16 (36.4)	0.629
	PR+	229 (59.6)	23 (67.6)		220 (59.5)	28 (63.6)	
HER2 status	HER2–	294 (77.0)	24 (70.6)	0.403	276 (75.0)	38 (86.4)	0.132
	HER2+	88 (23.0)	10 (29.4)		92 (25.0)	6 (13.6)	
Surgery	Breast conserving	273 (70.9)	16 (47.1)	0.006	264 (71.2)	23 (52.3)	0.015
	Mastectomy	112 (29.1)	18 (52.9)		107 (28.8)	21 (47.7)	
Chemotherapy	FEC-D	193 (50.1)	18 (52.9)	0.858	186 (50.1)	22 (50.0)	1.000
	FEC-DG	192 (49.9)	16 (47.1)		185 (49.9)	22 (50.0)	
Bisphosphonates	Zometa 2 years	193 (50.1)	17 (50.0)	1.000	185 (49.9)	23 (52.3)	0.874
	Zometa 5 years	192 (49.9)	17 (50.0)		186 (50.1)	21 (47.7)	
Circulating tumor cells (CTCs)	CTC– before chemo	316 (82.1)	20 (58.8)	0.003	303 (81.7)	32 (72.3)	0.160
	CTC+ before chemo	69 (17.9)	14 (41.2)		68 (18.3)	12 (27.7)	
	CTC– after chemo	304 (79.0)	27 (79.4)	1.000	302 (81.4)	28 (63.6)	0.009
	CTC+ after chemo	81 (21.0)	7 (20.6)		69 (18.6)	16 (36.4)	

EFC#93 serum DNAme was deemed positive (+ve) at or above a pattern frequency of 0.00008

^a^Two-sided t-test (contingent upon age) or Chi-square test (for all other parameters)

Information on N, PR and HER2 is missing from 1, 1 and 3 patients, respectively. Serum DNAme was not analysed for 4 post-treatment samples

*FEC-D* fluorouracil-epirubicin-cyclophosphamide (500/100/500 mg/m^2^, FEC) followed by docetaxel (100 mg/mg^2^), *FEC-DG* fluorouracil-epirubicin-cyclophosphamide (500/100/500 mg/m^2^, FEC) followed by gemcitabine (1000 mg/m^2^ d1,8)-docetaxel (75 mg/m^2^), *SD* standard deviation

**Table 2:** Univariate and multivariable proportional hazards model for relapse-free and overall survival for SUCCESS serum samples

Characteristic	Univariate analyses			
	Relapse-free survival		Overall survival	
	HR (95% CI)	*P* value	HR (95% CI)	*P* value
Menopausal status, post vs pre	1.323 (0.750–2.333)	0.335	2.872 (1.164–7.086)	0.022
Tumor size, T2-T4 vs T1	2.268 (1.187–4.332)	0.013	3.881 (1.343–11.218)	0.012
Lymph node involvement, N1-3 vs N0	1.645 (0.861–3.142)	0.132	3.012 (1.045–8.683)	0.041
Estrogen receptor (ER) status, ER- vs ER+	1.316 (0.999–1.734)	0.051	1.333 (0.918–1.934)	0.131
Progesterone receptor (PR) status, PR- vs PR+	1.180 (0.897–1.554)	0.237	1.219 (0.839–1.772)	0.298
HER2 status, HER2+ vs HER2-	1.907 (0.858–4.241)	0.113	1.789 (0.618–5.178)	0.283
Grading, G3 vs G1/2	1.079 (0.623–1.868)	0.786	1.129 (0.535–2.384)	0.75
CTCs before chemo, CTC+ vs CTC-	3.666 (2.110–6.368)	<0.0001	5.681 (2.686–12.014)	<0.0001
CTCs after chemo, CTC+ vs CTC-	1.401 (0.757–2.592)	0.283	1.467 (0.646–3.331)	0.36
EFC#93 before chemo, EFC#93+ vs EFC#93-	4.912 (2.613–9.233)	<0.0001	7.689 (3.518–16.804)	<0.0001
EFC#93 after chemo, EFC#93+ vs EFC#93-	1.913 (0.927–3.949)	0.079	1.807 (0.673–4.853)	0.24
	Multivariable analyses			
	Relapse-free survival		Overall survival	
	HR (95% CI)	*P* value	HR (95% CI)	*P* value
Menopausal status	1.294 (0.728–2.302)	0.379	2.688 (1.070–6.750)	0.035
Tumor size	1.763 (0.914–3.401)	0.091	2.945 (1.009–8.597)	0.048
Lymph node involvement	1.442 (0.750–2.775)	0.273	2.242 (0.765–6.566)	0.141
CTCs before chemo	2.847 (1.613–5.024)	0.0003	3.623 (1.681–7.812)	0.001
EFC#93 before chemo	3.782 (1.965–7.281)	<0.0001	5.973 (2.634–13.542)	<0.0001

Cox proportional hazards models. All statistical tests were two-sided

*CI* confidence interval, *CTC* circulating tumor cell, *HR* hazard ratio

**Fig. 4:**
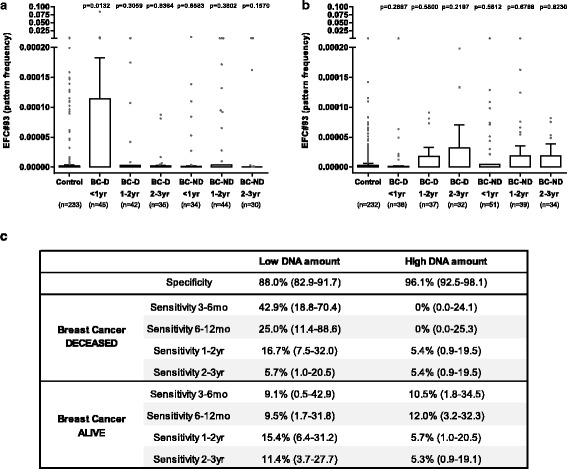
Pattern frequency of EFC#93 in women from the UKCTOCS. EFC#93 pattern frequency in samples with low (**a**) or high (**b**) amounts of DNA in the serum sample. **c** Performance of EFC#93 serum DNAme marker (cut-off threshold = 0.00008) depending on time to diagnosis and whether or not women subsequently died. Data separated based on DNA amount in the serum sample (95% CI in brackets). *P* values in (**a**) and (**b**) are from a Mann–Whitney U-test and are relative to the control group. *Control* no cancer developed, *BC-D* breast cancer which eventually led to death, *BC-ND* breast cancer which did not lead to death, *mo* months, *yr* years

To assess whether EFC#93 serum DNAme can diagnose women with poor prognostic BC earlier, we analyzed serum samples from 925 women from our UKCTOCS cohort. The amount of DNA as well as the fragment length was dramatically higher than expected and correlated with the average UK temperature (Additional file 1: Figures S11 and S12); there was also a good correlation between DNA amount and fragment length (Additional file 1: Figure S13) indicating a substantial leak of blood cell DNA into the serum during the blood transport. Within this nested case/control setting, the women with BC (cases) had provided serum samples up to three years before diagnosis. Again, we a priori hypothesized that the high background levels of DNA from lysed blood cells would impact on assay sensitivity, particularly in a pre-clinical setting where only traces of cancer DNA were expected in the circulation. We therefore split all samples into two groups: (1) low serum DNA amount; and (2) high serum DNA amount. In the “low DNA” group, we observed a significantly higher EFC#93 serum DNAme pattern frequency in the women who developed BC within one year after sample donation and subsequently died (Fig. 4a; cut-off threshold of 0.00008). Due to the high levels of background DNA, no significant findings were observed in the “high DNA” sample groups (Fig. 4b). In the “low DNA” group, EFC#93 DNAme was able to identify 43% of women 3–6 months and 25% of women 6–12 months before the diagnosis of a BC which eventually led to death, with a specificity of 88% (Fig. 4c). The sensitivity of serum EFC#93 methylation in detecting fatal BCs up to one year in advance of diagnosis was ~ 4-fold higher compared to non-fatal BCs (33.9% compared to 9.3%). In fact, the sensitivity for non-fatal BCs was within the false-positive range of the healthy samples, indicating that non-fatal BCs are not detected with this marker.

## Discussion

We demonstrate that our serum DNAme marker, EFC#93, can be detected up to one year in advance of BC diagnosis and is a marker for poor prognosis in the adjuvant primary treatment setting. EFC#93 is located within *GP5*, a gene coding for a surface glycoprotein which has been suggested to be involved in hematogenous breast cancer metastasis [36].

The use of tumor-specific methylated DNA in serum using targeted ultra-high bisulfite sequencing has the following advantages compared to alternative strategies: (1) patient plasma/serum DNA can be amplified to increase assay sensitivity; (2) abnormal DNAme is a stable tumor-specific marker occurring early in carcinogenesis and is conserved throughout disease progression [22]; (3) selection of CpG island hypermethylation simplifies assay design; and (4) DNAme over several linked CpGs constitutes a clearly detectable signal with a higher specificity (due to alleviated sensitivity to sequencing errors).

A key limitation of any current large-scale population-based cell-free DNA study, such as ours, is the lack of high-quality samples. This was evident in both the SUCCESS and UKCTOCS samples, where the blood samples were not processed until 24–96 h after the blood was drawn and hence contained large amounts of leaked WBC DNA. In healthy individuals, cell-free DNA is normally present at concentrations in the range of 0–100 ng/mL and at an average of 30 ng/mL [37]. DNA derived from tumor cells is also shorter than that from non-malignant cells in the plasma of cancer patients and typically 166 bp long [38]. Blood tubes which stabilize cell-free DNA and prevent leakage of WBC DNA are now available [39] and will be used for any future studies.

The leaked DNA in these serum samples will no doubt have led to a preferential amplification of non-cancer DNA. Despite these complicating factors, EFC#93 serum DNAme, before treatment, was a strong prognostic factor and was complementary to CTCs. Some previous studies on CTCs used a cut-off value of > 5 cells/mL; this may certainly be valid and useful for metastatic BC patients. In the SUCCESS setting of primary BC patients, only 8/419 patients (1.9%) had > 5 CTCs/mL. Had we taken this CTC cut-off, the relapse-free survival HR would have been 4.8 with a relatively wide 95% CI of 1.5–15.5 (*P* = 0.009). Hence, the chosen threshold that we pre-specified in previous work [10] (i.e. CTCs detectable or not) is completely justified in this primary cancer setting.

For the current genetic cell-free DNA markers the detection limit is in the range of 0.1% allele frequency (i.e. 1 mutated in the background of 1000 non-mutated alleles can be detected [15, 21]). Ultra-high coverage bisulfite-sequencing, however, allows for far more sensitive testing. Mammography screening in women aged 50–75 years has a sensitivity of 82–86% and a specificity of 88–92% for detecting any BC; however, the majority of these cancers are not fatal [40]. EFC#93 serum DNAme has a sensitivity of 43% in identifying fatal breast cancer up to six months in advance of current diagnosis at a similar specificity (88%) to mammography, supporting the rationale for incorporating serum DNAme markers in future cancer-screening trials.

Based on the evidence accumulated so far, we have to assume that EFC#93 indicates the presence of disseminated breast cancer, which at least in a proportion of women, will not yet be clinically evident in the breast. Hence, the question arises whether EFC#93-positive mammography-negative women should watch and wait (i.e. within an enhanced surveillance program) or whether this group of women could also be offered a strategy which actively deals with the likely disseminated disease until radiological evidence in the breast starts to arise. Anti-hormonal treatment (i.e. Tamoxifen or aromatase inhibitors) are being used for both adjuvant and preventive treatment. Therefore, we assessed whether EFC#93 positivity after SUCCESS chemotherapy (which is before the initiation of anti-hormonal treatment) is associated with survival: EFC#93 positivity in post-chemotherapy samples of hormone receptor-negative women still indicated a poor prognosis whereas EFC#93 positivity in hormone receptor-positive women was no longer associated with poor prognosis (Additional file 1: Figure S14). CTC status in post-chemotherapy samples was not associated with outcome irrespective of subsequent anti-hormonal treatment (Additional file 1: Figure S15).

## Conclusions

Overall and for the first time, our study provides evidence that serum DNAme markers can diagnose fatal BCs up to one year in advance of current diagnosis and enable individualized BC treatment which may even commence before obtaining radiological evidence in the breast. In addition, the combination of CTC and cell-free DNA analysis might further improve risk stratification of breast cancer patients. The recent advance of purposed blood tubes will facilitate clinical implementation of DNAme pattern detection of cell-free DNA as a clinical tool in cancer medicine.

## Additional files

Additional file 1: Figure S1. Samples from the SUCCESS trial analyzed within this study. **Figure S2**. Samples from the UKCTOCS cohort analyzed within this study (nested case/control setting). **Figure S3**. Absolute pattern counts for all patterns detected in the region of marker EFC#93 in Serum Set 1 samples. **Figure S4**. Pattern frequency of EFC#93 serum DNAme in two prospectively independently collected cohorts. **Figure S5**. DNA amount per mL serum in the prospectively collected serum (Set 1 and 2), SUCCESS cohort, and UKCTOCS cohort. **Figure S6**. Pattern frequency for EFC#93 measured in SUCCESS serum set samples from women with no, 1–4 or ≥ 5 CTCs in the matched blood sample before (A) or after (B) chemotherapy. **Figure S7**. Impact of the presence (+ve, ≥ 1 cancer cell in blood sample) or absence (-ve) of CTCs on patient outcome. **Figure S8**. Impact of the presence (+ve, EFC#93 pattern frequency ≥ 0.00008) or absence (-ve) of serum DNA methylation in CTC + ve (≥1 cancer cell in pre-chemotherapy blood sample) or absence CTC-ve patients. **Figure S9**. Relapse-free and overall survival according to samples taken after chemotherapy. **Figure S10**. Relapse-free and overall survival according to samples taken after chemotherapy. **Figure S11**. Average serum DNA amount correlates with average UK temperature. **Figure S12**. Average serum DNA fragment size correlates with average UK temperature. **Figure S13**. Correlation of DNA fragment size and DNA amount. **Figure S14**. Overall survival of women whose samples were taken before and after chemotherapy and before anti-hormonal treatment in hormone receptor-negative and -positive SUCCESS participants. **Figure S15**. Overall survival of women whose samples were taken before and after chemotherapy and before anti-hormonal treatment in hormone receptor-negative and -positive SUCCESS participants. (PDF 2123 kb)

Additional file 2: Supplementary Material and Methods: Additional details of samples sets, methods and analyses. (PDF 279 kb)

Additional file 3: Table S1. Coordinates and primers used to amplify the identified target regions using bisulfite sequencing. (PDF 578 kb)

## Abbreviations

AUC: Area under the curve
BC: Breast cancer
bp: Base pairs
CTC: Circulating tumor cells
DNAme: DNA methylation
ROC: Receiver operating characteristics
WBC: White blood cell
