# Supplementary material for: Methylation patterns in serum DNA for early identification of disseminated breast cancer
Source: Genome Med. 2017 Dec 22;9:115. doi: 10.1186/s13073-017-0499-9 (PMC5740791; doi:10.1186/s13073-017-0499-9)
Supplement: Supplementary file 1 — Samples from the SUCCESS trial analyzed within this study. Figure S2. Samples from the UKCTOCS cohort analyzed within this study (nested case/control setting). Figure S3. Absolute pattern counts for all patterns detected in the region of marker EFC#93 in Serum Set 1 samples. Figure S4. Pattern frequency of EFC#93 serum DNAme in two prospectively independently collected cohorts. Figure S5. DNA amount per mL serum in the prospectively collected serum (Set 1 and 2), SUCCESS cohort, and UKCTOCS cohort. Figure S6. Pattern frequency for EFC#93 measured in SUCCESS serum set samples from women with no, 1–4 or ≥ 5 CTCs in the matched blood sample before (A) or after (B) chemotherapy. Figure S7. Impact of the presence (+ve, ≥ 1 cancer cell in blood sample) or absence (-ve) of CTCs on patient outcome. Figure S8. Impact of the presence (+ve, EFC#93 pattern frequency ≥ 0.00008) or absence (-ve) of serum DNA methylation in CTC + ve (≥1 cancer cell in pre-chemotherapy blood sample) or absence CTC-ve patients. Figure S9. Relapse-free and overall survival according to samples taken after chemotherapy. Figure S10. Relapse-free and overall survival according to samples taken after chemotherapy. Figure S11. Average serum DNA amount correlates with average UK temperature. Figure S12. Average serum DNA fragment size correlates with average UK temperature. Figure S13. Correlation of DNA fragment size and DNA amount. Figure S14. Overall survival of women whose samples were taken before and after chemotherapy and before anti-hormonal treatment in hormone receptor-negative and -positive SUCCESS participants. Figure S15. Overall survival of women whose samples were taken before and after chemotherapy and before anti-hormonal treatment in hormone receptor-negative and -positive SUCCESS participants. (PDF 2123 kb) [file 13073_2017_499_MOESM1_ESM.pdf]

Supporting information for:

Methylation Patterns in Serum DNA for Early Identification of Disseminated Breast Cancer

Supplementary Figures

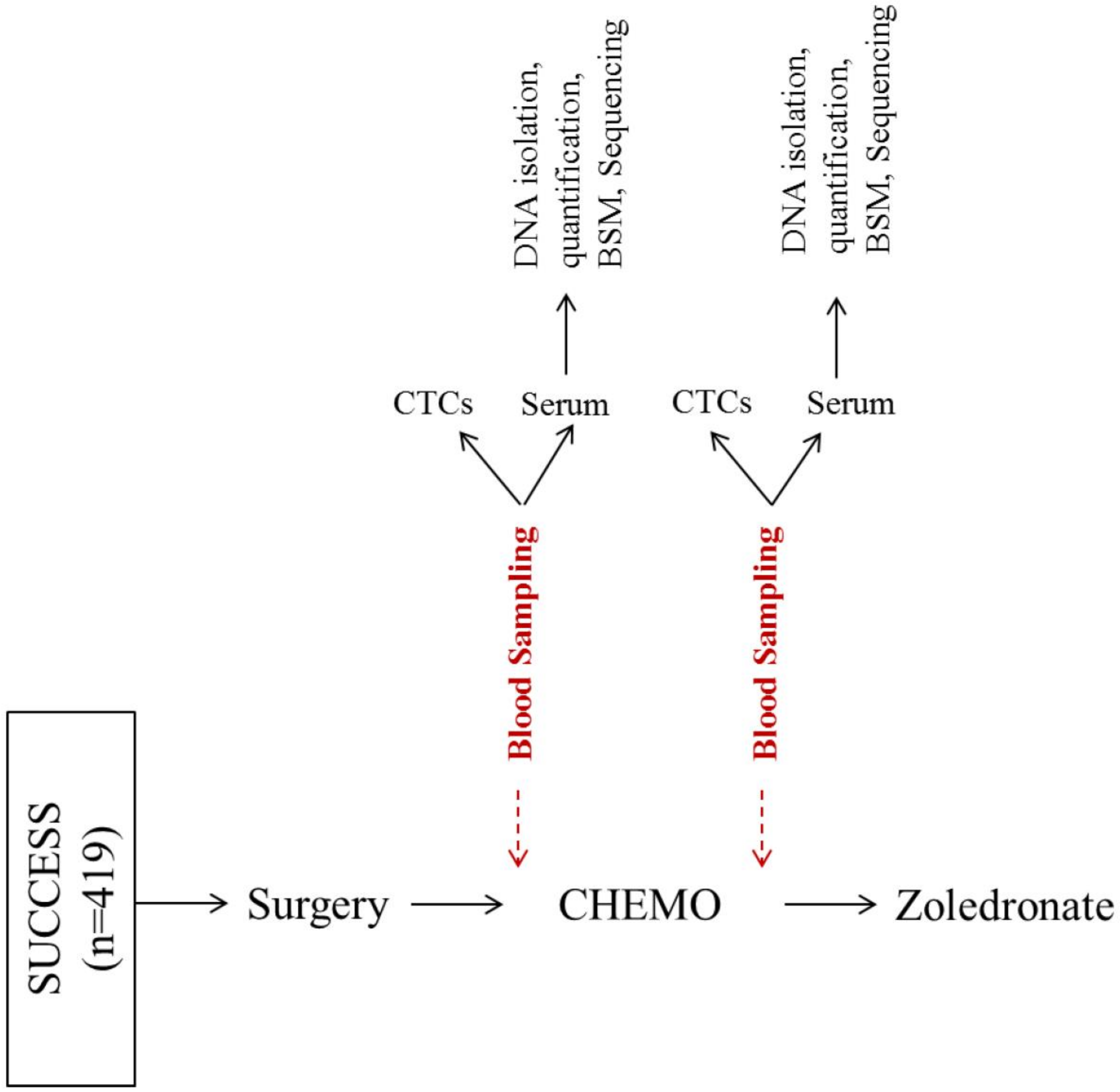

Fig S1 Samples from the SUCCESS trial analyzed within this study.

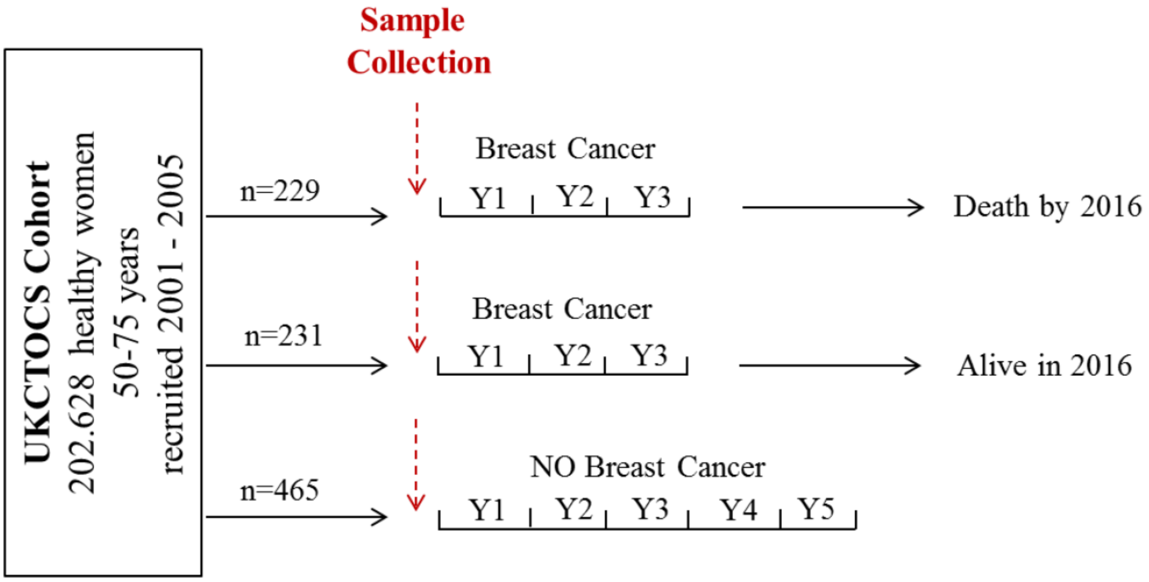

Fig S2 Samples from the UKCTOCS cohort analyzed within this study (nested case/control setting).

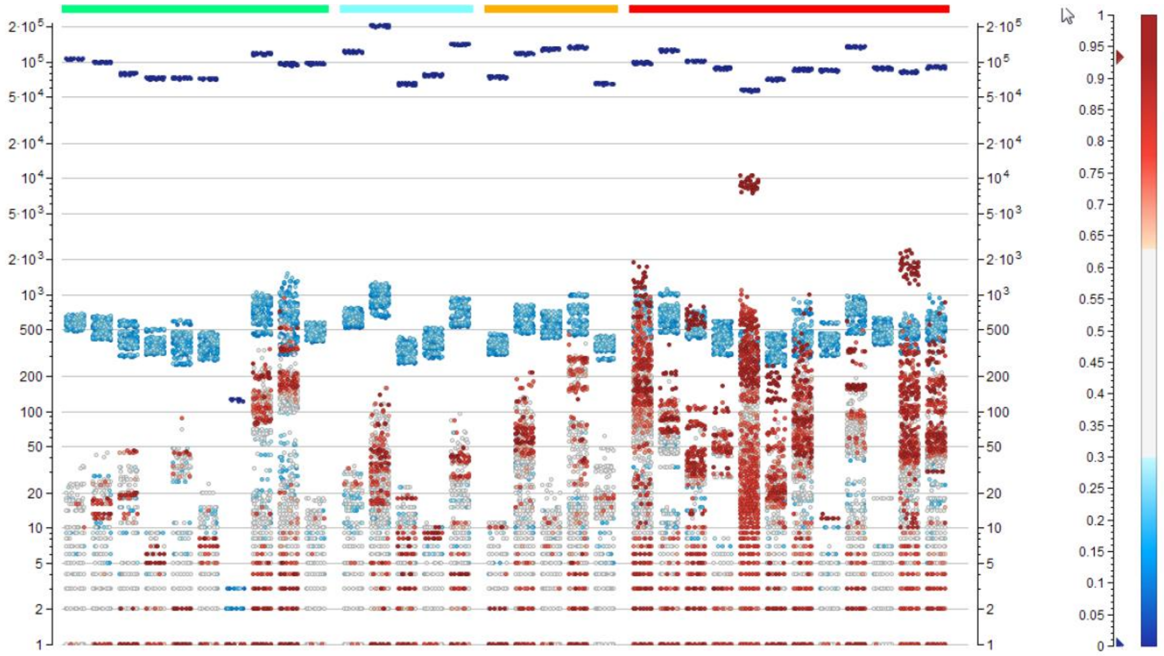

Fig S3 Absolute pattern counts for all patterns detected in the region of marker EFC#93 in Serum Set 1 samples. Column plot with individual dots for all patterns for each of the samples. The y-axis gives counts per pattern (log scale); samples are grouped into healthy (green), benign tumor (blue), primary breast cancer (orange), and metastatic breast cancer (red). Patterns are colored according to methylation level (color scale ranging from 0 to 1, with 1 = all CpGs of the pattern methylated). Patterns with similar methylation levels cluster together, for example fully methylated patterns have similar read counts, independently of the number of CpGs contained in a pattern (between 4 and 11 in this case) and which of the available CpGs are included. Fully un-methylated patterns are detected most frequently in all samples (>10^4 counts), while patterns with a single methylated CpG are observed about 20-fold less often, largely independent of the position of the methylated CpG. In samples where highly methylated patterns are observed, fully methylated patterns are counted more frequently than patterns containing one or two unmethylated CpGs. Dots representing highly methylated patterns are displayed in the foreground, while unmethylated patterns are in the background for visibility reasons (blue and white dots are about four times more abundant than red dots).

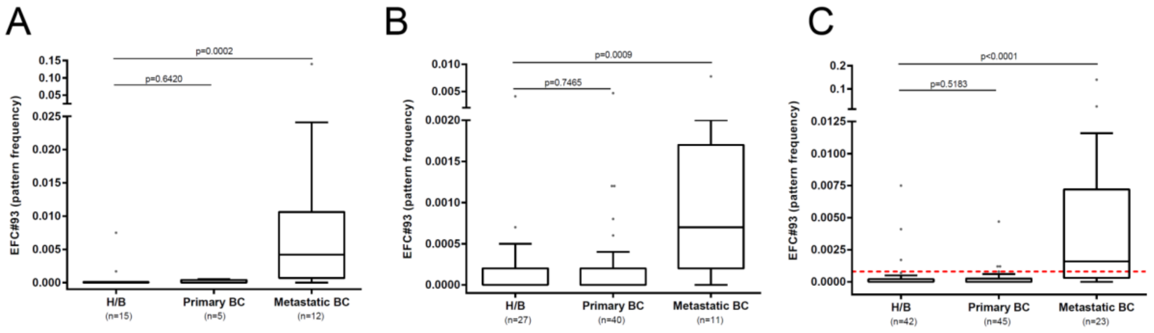

Fig S4 Pattern frequency of EFC#93 serum DNAme in two prospectively independently collected cohorts. Panel A represents Set 1 and panel B is Set 2. A cut-off threshold of 0.0008 was set when Sets1 and 2 data were combined (C). P values from a Mann-Whitney-U-test. H/B, Healthy/Benign; BC, Breast Cancer.

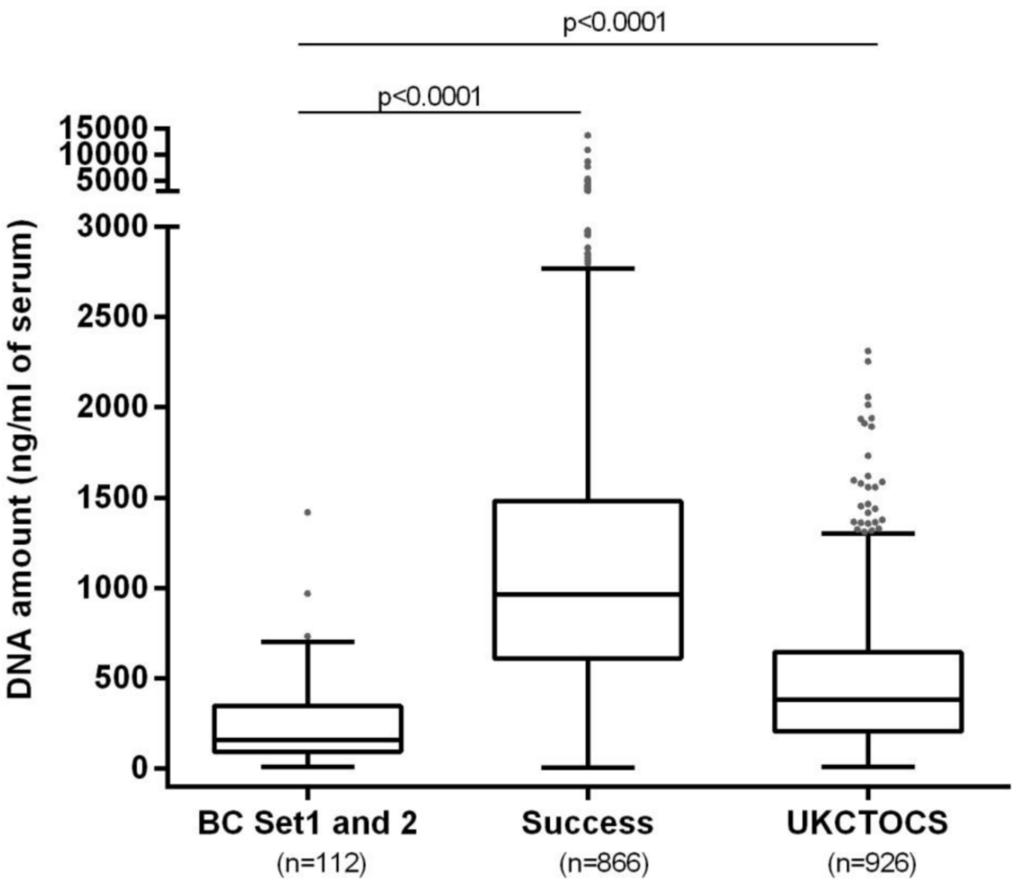

Fig S5 DNA amount per mL serum in the prospectively collected serum (Set 1 and 2), SUCCESS cohort, and UKCTOCS cohort. P values are based on a Mann-Whitney-U-test.

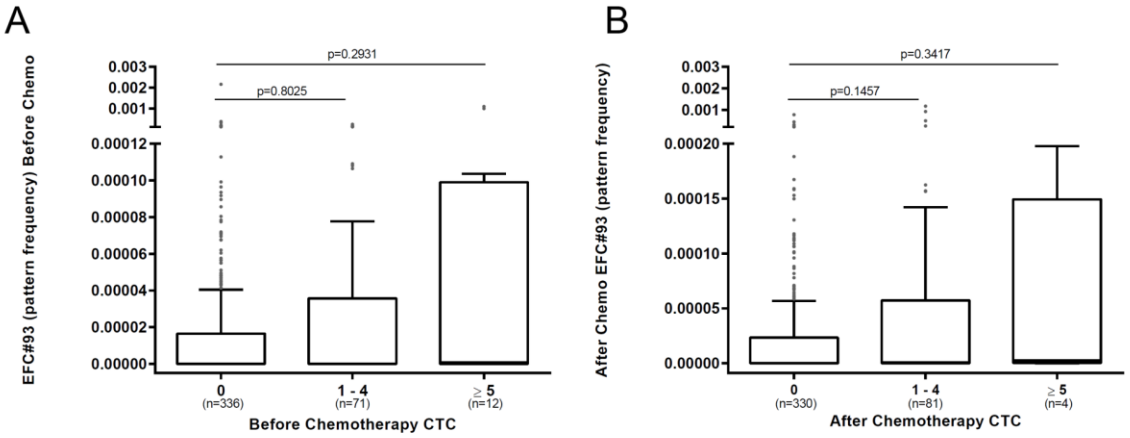

Fig S6 Pattern frequency for EFC#93 measured in SUCCESS serum set samples from women with no, 1-4 or ≥5 CTCs in the matched blood sample before (A) or after (B) chemotherapy. P values for a Mann-Whitney-U-test.

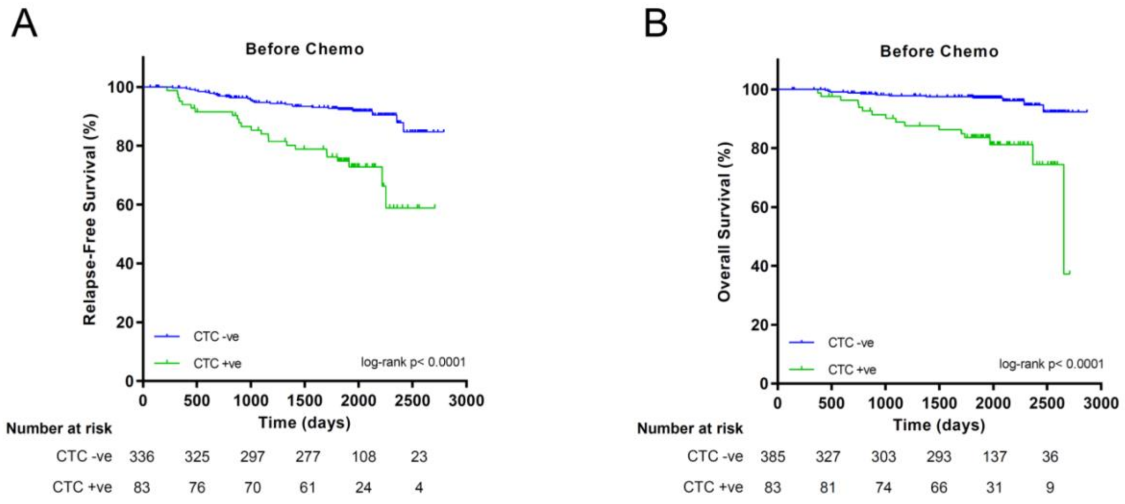

Fig S7 Impact of the presence (+ve, ≥1 cancer cell in blood sample) or absence (-ve) of CTCs on patient outcome. Two-sided log-rank test.

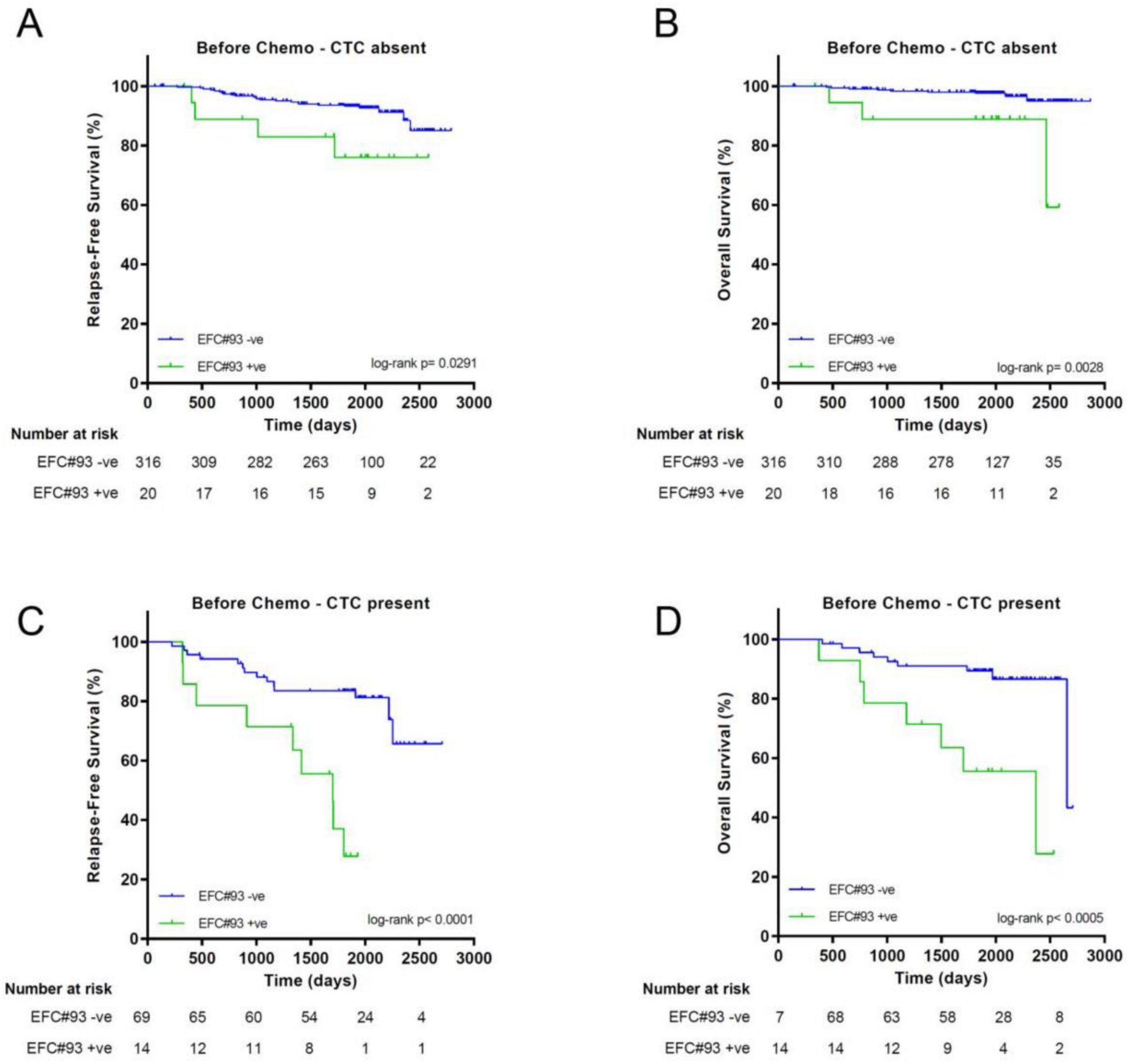

Fig S8 Impact of the presence (+ve, EFC#93 pattern frequency ≥ 0.00008) or absence (-ve) of serum DNA methylation in CTC +ve (≥1 cancer cell in pre-chemotherapy blood sample) or absence CTC-ve patients. Two-sided log-rank test.

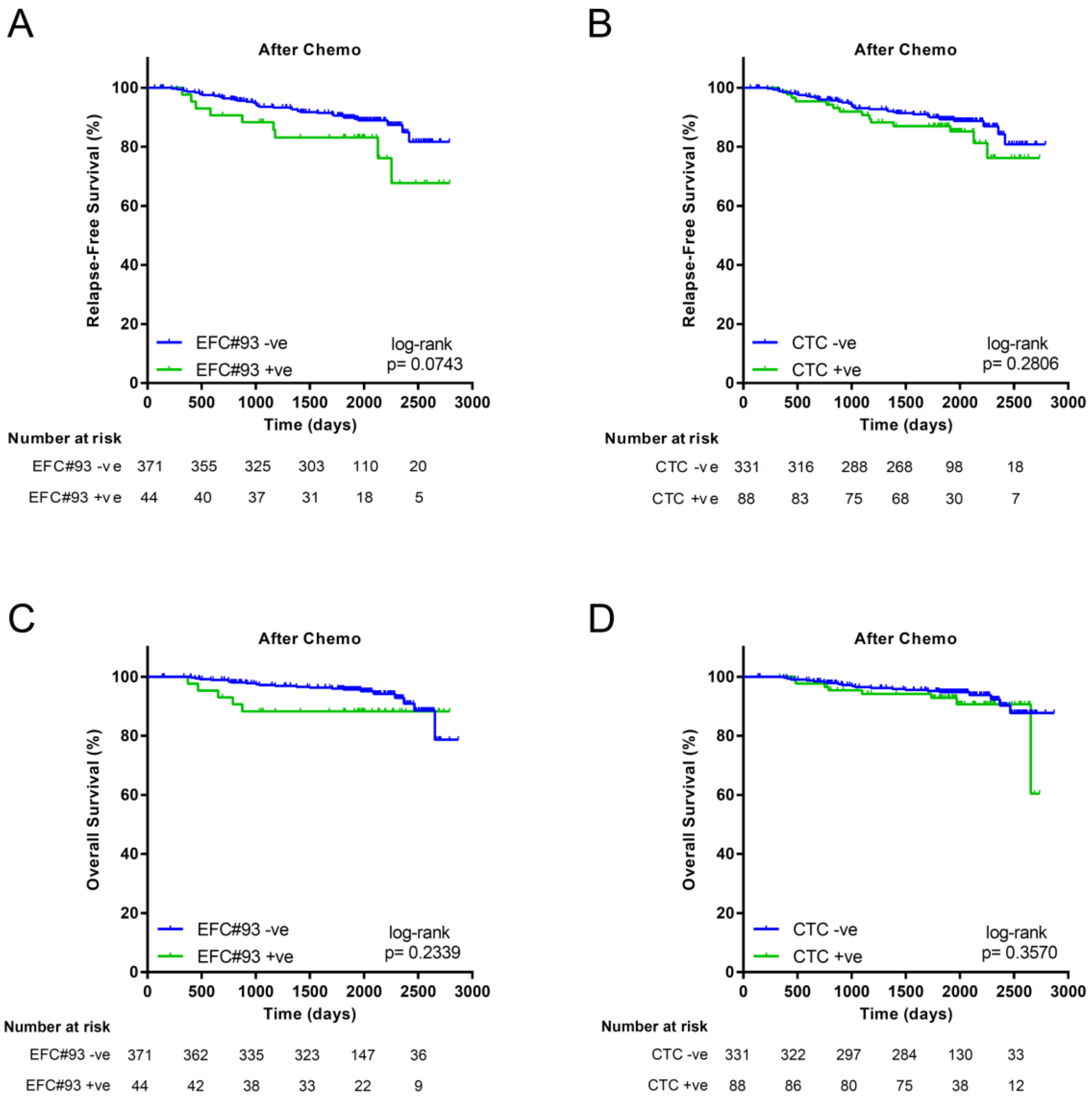

Fig S9 Relapse-Free survival (A, B) and Overall survival (C, D) according to samples taken after chemotherapy. Impact of the presence (+ve, EFC#93 pattern frequency ≥ 0.00008; ≥ 1 CTC) or absence (-ve) of EFC#93 methylation or CTC on patient survival. Two-sided log-rank test.

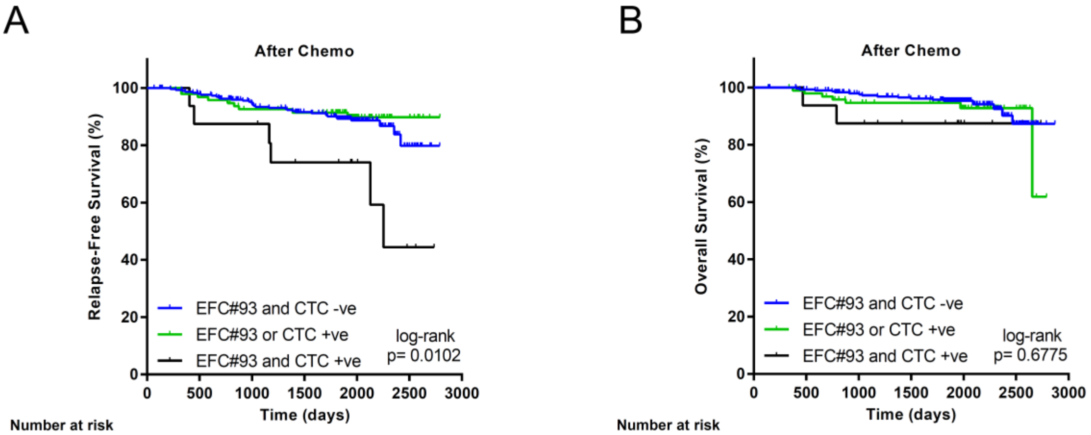

Number at risk (A)

EFC#93 and CTC -ve	302	290	265	247	87	16
EFC#93 or CTC+ve	97	93	85	79	37	9
EFC#93 and CTC +ve	16	15	15	11	7	3

Number at risk (B)

EFC#93 and CTC -ve	302	295	273	262	116	29
EFC#93 or CTC+ve	97	94	86	83	45	12
EFC#93 and CTC +ve	16	16	15	12	8	5

Fig S10 Relapse-Free survival (A) and Overall survival (B) according to samples taken after chemotherapy. Impact of the presence of both, one or the other or neither EFC#93 methylation and/or CTC presence on patient survival. Two-sided log-rank test.

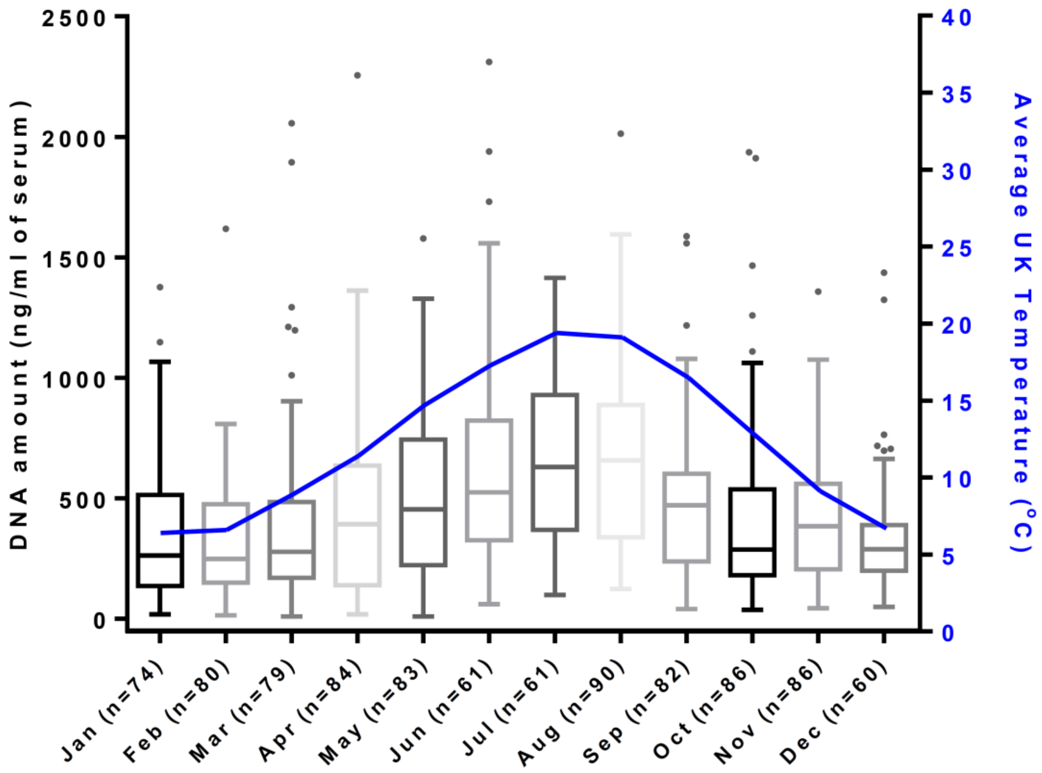

Fig S11 Average serum DNA amount correlates with average UK temperature. Boxplot of DNA amount extracted from UKCTOCS sample set, collected at certain months of the year. Blue line represents average monthly UK temperatures (average UK data from 1981-2010 data set; metoffice.gov.uk).

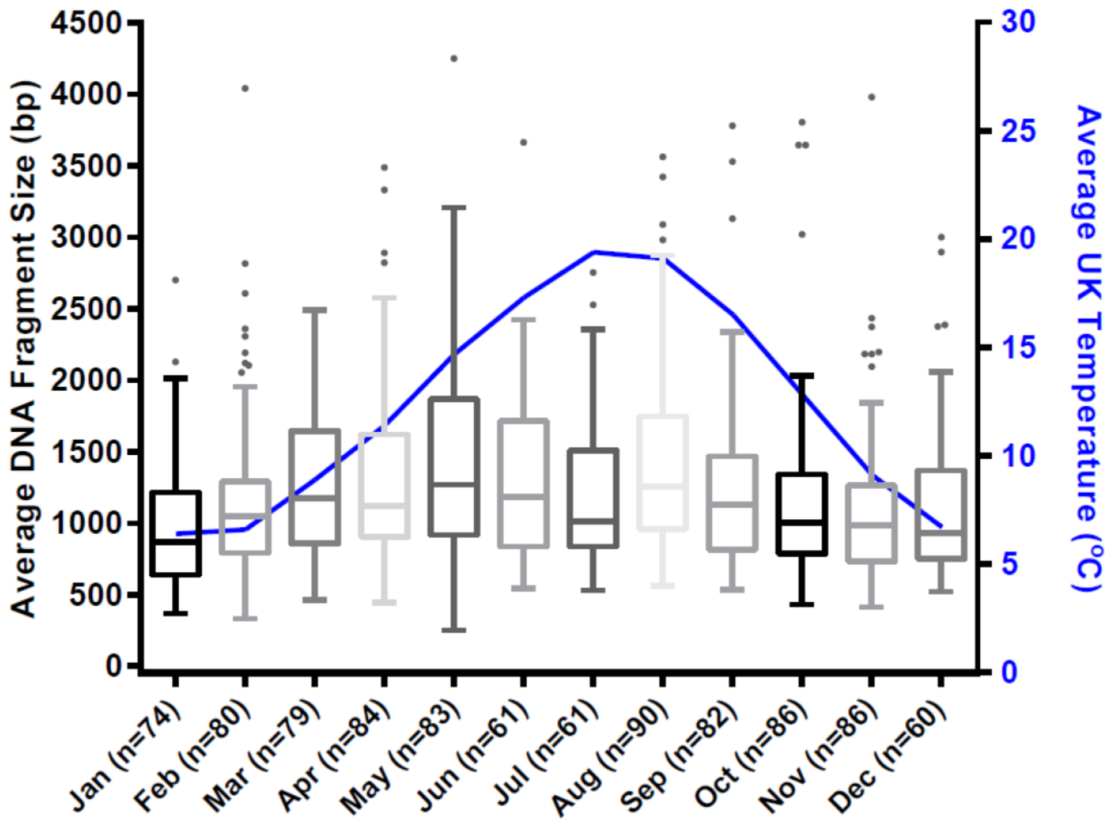

Fig S12 Average serum DNA fragment size correlates with average UK temperature. Boxplot of DNA fragment size of the DNA extracted from UKCTOCS sample set, collected at certain months of the year. Blue line represents average monthly UK temperatures (average UK data from 1981-2010 data set; metoffice.gov.uk).

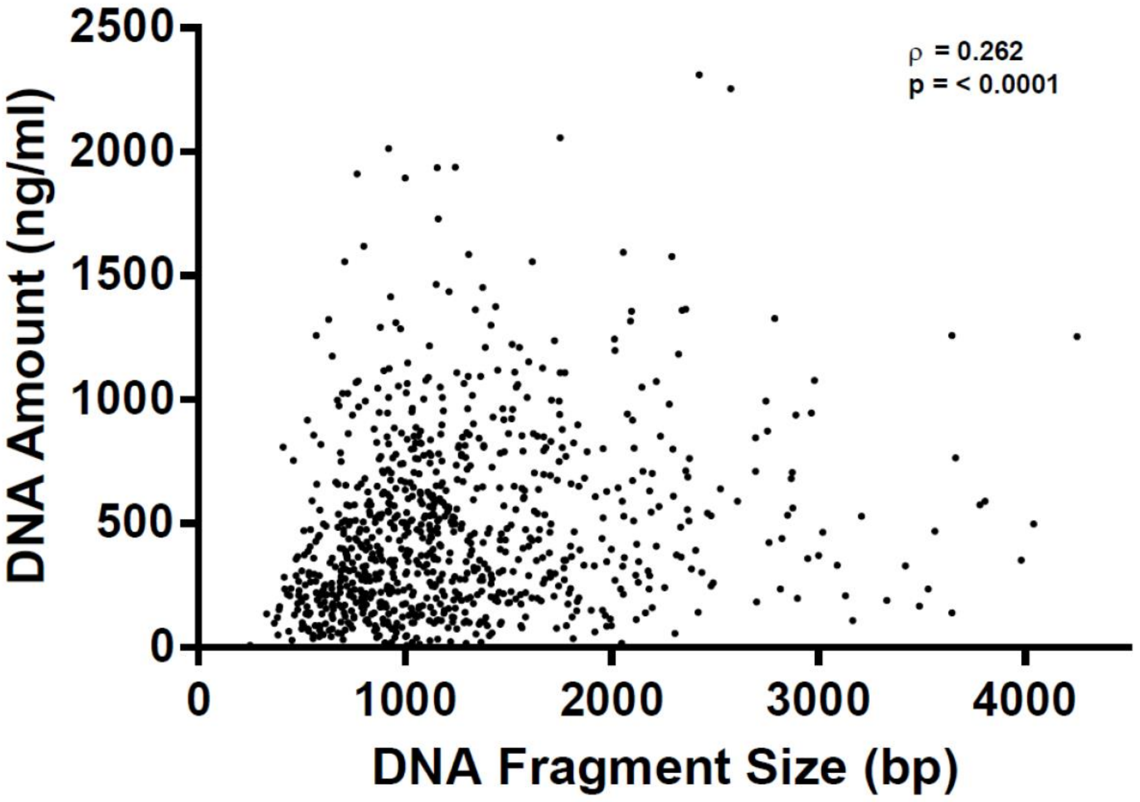

Fig S13 Correlation of DNA fragment size and DNA amount. Scatter-plot of DNA fragment size and DNA amount extracted from UKCTOCS sample set.

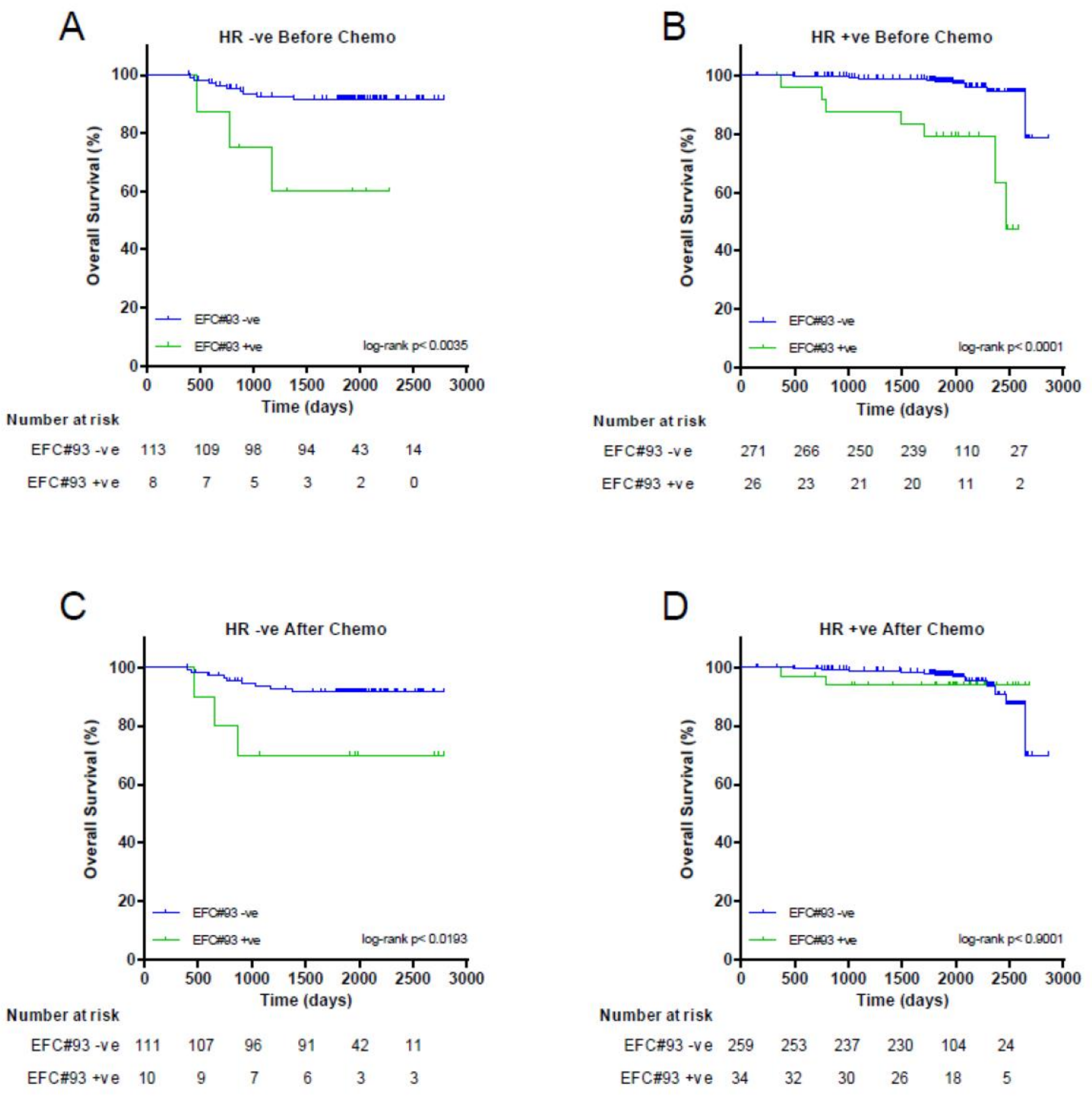

Fig S14 Overall survival of women whose samples were taken before (A, B) and after chemotherapy (C, D) and prior to anti-hormonal treatment (D) in Hormone-Receptor (HR) negative (A, C) and positive (B, D) SUCCESS participants. Impact of the presence (+ve, EFC#93 pattern frequency ≥ 0.00008) or absence (-ve) of EFC#93 methylation on patient overall survival. Two-sided log-rank test.

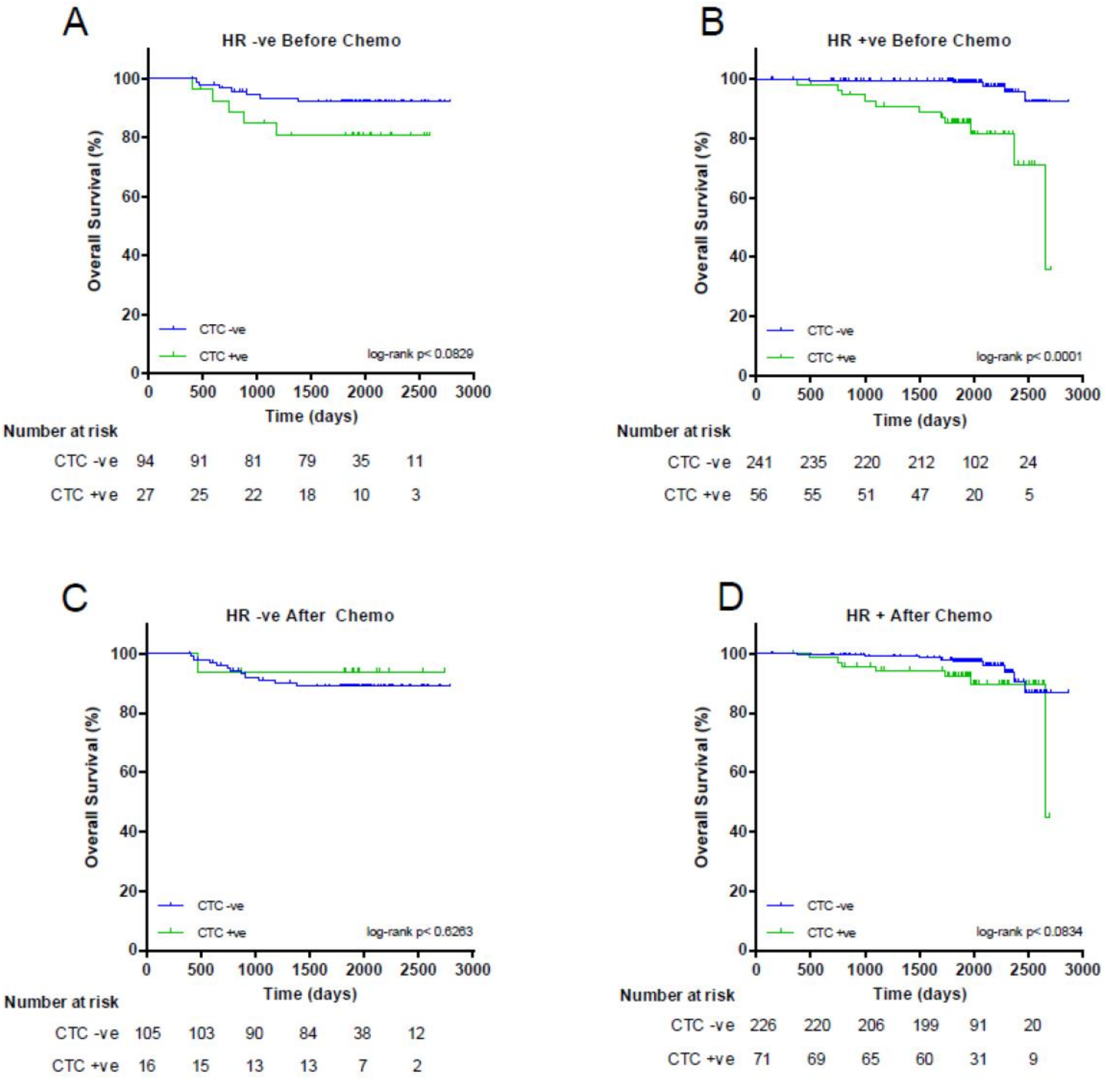

Fig S15 Overall survival of women whose samples were taken before (A, B) and after chemotherapy (C, D) and prior to anti-hormonal treatment (D) in Hormone-Receptor (HR) negative (A, C) and positive (B, D) SUCCESS participants. Impact of the presence (+ve, ≥ 1 CTC) or absence (-ve) of CTC on patient overall survival. Two-sided log-rank test.
