# Supplementary material for: Methylation patterns in serum DNA for early identification of disseminated breast cancer
Source: Genome Med. 2017 Dec 22;9:115. doi: 10.1186/s13073-017-0499-9 (PMC5740791; doi:10.1186/s13073-017-0499-9)
Supplement: Supplementary file 2 — Supplementary Material and Methods: Additional details of samples sets, methods and analyses. (PDF 279 kb) [file 13073_2017_499_MOESM2_ESM.pdf]

Supplementary Material and Methods: Additional details of samples sets, methods and analyses.

Subjects and sample collection:

We analyzed a total of 5 sets as detailed in Figure 1.

RRBS-Set:

Eight prospectively collected invasive ductal breast cancer samples (2/8 triple negative; mean age = 56.6 years), and twenty three white blood cell samples (mean age = 57.8) were assessed by RRBS. All samples were collected prospectively at the University College London Hospital in London (University College London Hospital, 235 Euston Rd, Fitzrovia, London NW1 2BU) and at the Charles University Hospital in Prague (Gynecological Oncology Center, Department of Obstetrics and Gynecology, Charles University in Prague, First Faculty of Medicine and General University Hospital, Prague, Apolinarska 18128 00 Prague 2, Czech Republic) and at the Department of Gynaecology and Obstetrics, Klinikum Innenstadt, Ludwig-Maximilians-Universitaet Muenchen, Maistr.11, 80337 Munich, Germany. The study was approved by the local research ethics committees: UCL/UCLH Biobank for Studying Health & Disease NC09.13), the ethics committee of the General University Hospital, Prague and by the ethical committee of the Ludwig-Maximilians-University Munich. All patients provided written informed consent.

Prospectively collected Serum Sets

<u>Set 1:</u>

Serum samples from the following volunteers were collected (at the time of diagnosis, prior to treatment):

- Healthy/Benign volunteers (n=15, mean age 40.2 years).
- Patients with primary breast cancer (n=5, mean age 51.4 years).

- Patients with metastatic (distant metastases) breast cancer (n=12, mean age 60.12 years).

Set 2:

Serum samples from the following volunteers were collected (at the time of diagnosis, prior to treatment):

- Healthy/Benign volunteers (n=27, mean age 42.4 years).
- Patients with primary breast cancer (n=40, mean age 59.6 years).
- Patients with metastatic (distant metastases) breast cancer (n=11, mean age 60.2 years).

All samples were collected prospectively at the University College London Hospital in London and at the Charles University Hospital in Prague and the Department of Gynaecology and Obstetrics, Klinikum Innenstadt, Ludwig-Maximilians-Universitaet Muenchen, Maistr.11, 80337 Munich, Germany. The study was approved by the local research ethics committees: UCL/UCLH Biobank for Studying Health & Disease NC09.13) and the ethics committee of the General University Hospital, Prague approval No.: 22/13 GRANT – 7. RP – EPI-FEM-CARE as well as by the ethical committee of the Ludwig-Maximilians-University Munich. All patients provided written informed consent.

SUCCESS Set:

SUCCESS was a prospective, randomized adjuvant study comparing three cycles of fluorouracil-epirubicin-cyclophosphamide (FEC; 500/100/500 mg/m2) followed by 3 cycles of docetaxel (100 mg/m2) every 3 weeks vs three cycles of FEC followed by 3 cycles of gemcitabine (1000 mg/m2 d1,8)-docetaxel (75 mg/m2) every 3 weeks. After chemotherapy completion, the patients were further randomized to receive either 2 or 5 years of zoledronate. Hormone receptor–positive women received adequate endocrine treatment. The research questions associated with CTC analysis, the blood sampling time points, and the methodology were prospectively designed, and the prognostic value of the CTCs was defined as a scientific objective of the study protocol.

The study was approved by 37 German ethical boards (lead ethical board: Ludwig-Maximilians-University Munich) and conducted in accordance with the Declaration of Helsinki.

Blood samples for CTC enumeration as well as storage of serum were collected from patients after complete resection of the primary tumor and before adjuvant chemotherapy after written informed consent was obtained. The samples were collected within a time interval of less than 96 hours between the blood collection and sample preparation. A follow-up evaluation after chemotherapy and before the start of endocrine or bisphosphonate treatment was available for a subgroup. A total of 419 women had blood samples taken at both times points (i.e. before and after chemotherapy), had their CTCs enumerated at both time points and had sufficient serum available at both time points. For further details see Rack et al (1).

UKCTOCS Set:

From the UK Collaborative Trial of Ovarian Cancer Screening (UKCTOCS) (2) all 229 women (among the 202,628 women recruited between 2001 - 2005) who developed BC in the first three years after serum sample donation and died subsequent to this at cancer/death registry follow-up by 25th March 2015 and 231 matched women who developed BC within three years after sample donation and were alive at the end of follow-up and 465 women who did not develop BC within five years after sample donation were analyzed (Appendix p 4, 8). Blood samples from all UKCTOCS volunteers were spun down for serum separation after having been transported at room temperature from trial centres to the central laboratory. The median time between sample collection and centrifugation of the sample set was 22.1 hours (IQR 19.7-24.3). Only 1 mL of serum per UKCTOCS volunteer was available. The study was approved by the local research ethics committees (UCL/UCLH Biobank for Studying Health & Disease NC09.13) and was approved as part of trial approval by the UK North West Multicentre Research Ethics Committees (North West MREC

00/8/34). All patients provided written informed consent. For further details see Jacobs, Menon et al (2).

DNA methylation analyses in tissue samples:

DNA was isolated from tissue samples using the Qiagen DNeasy Blood and Tissue Kit (Qiagen Ltd, UK, 69506) and 600ng was bisulfite converted using the Zymo methylation Kits (Zymo Research Inc, USA, D5004/8).

Reduced Representation Bisulfite Sequencing (RRBS):

RRBS libraries were prepared by GATC Biotech using INVIEW RRBS-Seq according to proprietary SOPs. In brief, DNA was digested with the restriction endonuclease MspI that is specific for the CpG containing motif CCGG; later a size selection provides enhanced coverage for the CpG-rich regions including CpG islands, promoters and enhancer elements (3;4). The digested DNA is then adapter ligated, bisulfite modified and PCR-amplified. The libraries were sequenced on Illumina's HiSeq 2500 with 50 bp or 100 bp paired-end mode. After sequencing raw data was trimmed using Trimmomatic (0.32) to remove adapter sequences and low quality bases at the beginning and end of reads. Subsequently, reads were trimmed with TrimGalore (0.3.3) to remove cytosines derived from library preparation which must not be included in the methylation analysis. Read pairs were mapped to the human genome (hg19) in Genedata Expressionist® for Genomic Profiling 8.0 applying Bisulfite Mapper based on BOWTIE v2.1.0 (5) with the settings --no-discordant --reorder -p 8 --end-to-end --no-mixed -D 50 -k 2 --fr --norc -X 400 -I 0 --phred33. Further analysis was carried out using Genedata Expressionist® for Genomic Profiling 9.1.

Computation of methylation pattern frequencies:

In order to allow the sensitive detection of low-abundant methylation patterns, the read data available for each sample type (i.e. breast cancer and white blood cells) was pooled across patients and sequencing runs. Candidate genomic regions for methylation pattern analysis

were defined based on bundles of at least 10 paired-end reads covering at least 4 consecutive CpG sites which are located within a genomic range of at most 150bp. As illustrated in the figure below, our algorithm first determines sets of consecutive CpG sites of maximum size, from which multiple potentially overlapping subsets are derived, which still meet the selection criteria. CpG sites located in the gap between the mate reads are ignored. For each derived set of CpG sites, the absolute and relative frequencies of all methylation patterns observed in the corresponding reads are determined. The methylation patterns are represented in terms of binary strings in which the methylation state of each CpG site is denoted by 1 if methylated or 0 if unmethylated. The algorithm for selecting candidate regions and calculating methylation pattern frequencies was implemented in our software platform Genedata Expressionist® for Genomic Profiling.

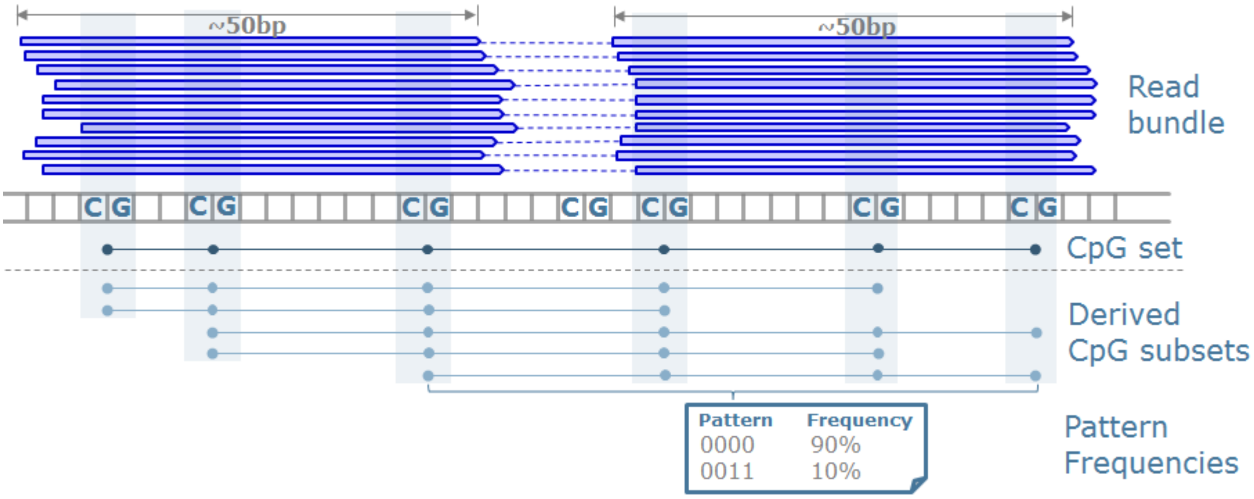

Procedure for the selection of tumor-specific patterns:

In order to ensure that the pattern exclusively occurs in tumor samples, all patterns present in white blood cells were excluded. A score for assessing the relevance of each pattern was determined by integrating multiple subordinate scores which quantitatively capture desired properties of candidate biomarker patterns. First, for each pattern a Tumor Specificity Score $S_P = DL \cdot TP \cdot TE \cdot AF$ was calculated, which consists of the four components Dilution Factor *DL*, Tumor Prevalence *TP*, Tumor Enrichment Factor *TE* and Avoiding Factor *AF*. The formal definitions of the score components are given in the following:

$$DL_{WBC} = \frac{\#total\ reads}{\#reads\ with\ pattern} * \frac{1}{10^3}$$
$$TP_{tumor} = \frac{\#reads\ with\ pattern\ in\ tumor}{\#total\ reads\ in\ tumor} * 10$$
$$TE_{tumor} = \frac{\#observed\ reads\ with\ pattern\ in\ tumor}{\#expected\ reads\ with\ pattern\ in\ tumor}$$
$$AF_{WBC} = \frac{\#expected\ reads\ with\ pattern\ in\ WBC}{\#observed\ reads\ with\ pattern\ in\ WBC}$$

The Dilution Factor *DL* and Tumor Prevalence *TP* favor patterns which are supported by a high proportion of reads in tumor and low proportion of reads in WBC, respectively. A pattern observed in 1 out of 10 reads in tumor and in 1 out of 1000 reads in WBC scores 1 for both factors. The Tumor Enrichment Factor *TE* and Avoiding Factor *AF* were included to assess the overrepresentation of the pattern in tumor samples and its underrepresentation in WBC samples, respectively, relative to an expected number of pattern reads which is based on the observed overall methylation level in those tissues. In order to estimate the number of expected reads supporting the pattern, the methylation frequencies are calculated for each CpG site individually. Next, the number of expected reads with a specific pattern is calculated as the product of the relative frequencies of the tumor specific methylation states observed for each CpG site in the pattern times the number of reads stretching across the pattern. A TE >1 indicates that a pattern is more frequent in tumor than expected when randomly distributing the observed methylation levels across reads. Besides favoring tumor specificity our scoring procedure was also designed to make patterns with high variance of the highest priority (i.e. patterns for which a high number of transitions in the methylation state is observed between consecutive CpG sites). Such patterns may be a product of the epigenetic reprogramming of tumor cells and in order to account for the potentially increased biological relevance of these patterns another score component was introduced. The normalized variance V_P of a pattern *p* is defined as the pattern variance divided by the maximum variance, i.e. the pattern length minus 1. The scores for the tumor specificity S_P and pattern variance V_P were combined in the tumor-specific variance score $SV_P =$

$V_P \cdot \log(S_P)$. In order to facilitate the ranking of each candidate genomic region *r* based on the relevance of patterns $p_1, \ldots, p_N$ observed in the region the aggregation score AS_r was calculated based on the following formula:

$$AS_r = \sum_{i=1}^{n} \frac{1}{i} SV_{Pi}$$

The aggregation score AS_r corresponds to a weighted sum of the tumor-specific variance scores of the observed patterns. The weighting was included since an ordinary sum would introduce a bias towards regions, in which a high number of patterns have been observed due to a high read coverage and/or high CpG site density. All of the presented statistics for assessing the relevance of methylation patterns and genomic regions were implemented in Genedata Expressionist® for Genomic Profiling and R, respectively.

DNA methylation analyses in serum samples:

Serum separation:

For Serum Sets 1-3 and the NACT Serum Set, women attending the hospitals in London and Prague were invited, consented and 20-40 mL blood obtained (VACUETTE® Z Serum Sep Clot Activator tubes, Cat 455071,Greiner Bio One International GmbH), centrifuged at 3,000rpm for 10 minutes and serum collected and stored at -80°C. We applied non-stringent measures (i.e. allowed for up to 12 hours between blood draw and centrifugation) purposely in order to mimic the situation of UKCTOCS samples which were sent from the recruiting centre to UCL within 24-48 hours before centrifugation.

Serum DNA isolation and bisulfite modification:

DNA was isolated at GATC Biotech (Konstanz, Germany). Serum DNA was quantified using the Fragment Analyzer and the High Sensitivity Large Fragment Analysis Kit (AATI, USA). DNA was bisulfite converted at GATC Biotech.

Targeted ultra-high coverage bisulfite sequencing:

Targeted bisulfite sequencing was performed at GATC Biotech. To this end, a two-step PCR approach was used similar to the recently published BisPCR2 (6). Bisulfite modification was performed with 1 mL serum equivalent. For each batch of samples positive and non-template controls were processed in parallel. Bisulfite converted DNA was used to test up to three different markers using automated workflows. After bisulfite modification the target regions were amplified using primers carrying the target specific sequence and a linker sequence. Amplicons were purified and quantified. All amplicons of the same sample were pooled equimolarly. In a second PCR, primers specific to the linker region were used to add sequences necessary for the sequencing and multiplexing of samples. Libraries were purified and quality controlled. Sequencing was performed on Illumina's MiSeq or HiSeq

2500 with 75 bp or 125 bp paired-end mode. Trimming of adapter sequences and low quality bases was performed with Trimmomatic as described for the RRBS data.

Assessment of pattern frequency in serum DNA:

After sequencing, raw data was trimmed using Trimmomatic (0.32) to remove adapter sequences and low quality bases at the beginning and end of reads. Subsequently, reads were trimmed with TrimGalore (0.3.3) to remove cytosines derived from library preparation which must not be included in the methylation analysis. Further analysis was carried out using Genedata Expressionist® for Genomic Profiling 9.1. Read pairs were mapped to the human genome (hg19) applying Bisulfite Mapper based on BOWTIE v2.2.5 (5) with the settings --no-discordant -p 8 --norc --reorder -D 50 --fr --end-to-end -X 500 -I 0 --phred33 -k 2 --no-mixed. Coverage was calculated per sample and target region using Numeric Data Feature Quantification activity by calculating the arithmetic mean of the coverage in each region. As part of the data quality control, efficiency of the bisulfite conversion was estimated in each sample by quantifying the methylation levels of CpHpG and CpHpH sites (where H is Any Nucleotide Except G), with minimum coverage of 10, within the target regions. Methylation pattern frequencies in serum samples for target regions were determined as described above.
