# Supplementary material for: Methylation patterns in serum DNA for early identification of disseminated breast cancer
Source: Genome Med. 2017 Dec 22;9:115. doi: 10.1186/s13073-017-0499-9 (PMC5740791; doi:10.1186/s13073-017-0499-9)
Supplement: Supplementary file 3 — Coordinates and primers used to amplify the identified target regions using bisulfite sequencing. (PDF 578 kb) [file 13073_2017_499_MOESM3_ESM.pdf]

Supplementary Tables

#	Coordinates	Primer sequence 1	Primer sequence 2	Amplicon size
89	chr1:3157511-3157650	GGGGTGYGGGGAGGTTGAGA	TCCCCRACTCCCCCAACCTC	140
91	chr2:19550330-19550456	GGYGTTGAAGTTGGAGAGGTTATTTTG	CCRAAACTCTTCTCCTTAAAACAAAAC	127
92	chr2:19550279-19550427	GGTTTTTTTTAGTTTTAGYGTTTTGA	TACAACAAAAAAACTTATAATCCAATTATCATC	149
93	chr3:194118853-194118957	YGTGAGGTTGGTGGGTAGGTTTAG	TTCCCCTATCCRCCAACTTACAAATATATCTTC	105
94	chr3:194118827-194118950	GTTYGTTAGTTTGTAAGTGTGTTTTT	CRACCCATTCCRAAAAACAAAATATA	124
95	chr3:128712373-128712480	GAATAATAGATAAGGGTGGTTGGTAGTAAGTA	TCACCTAAAACAAACATTCCAAAAACC	108
96	chr3:128712370-128712482	GTTTATTTGGGGTAGGTATTTTAGAAGTT	AAAAAACAACAAATAAAAATAACTAACAATAAACA	113
97	chr4:139483017-139483134	TYGGGATAGTATTTTGGGAGTTGGG	ACCAAACRCCCATCCAATTTACCTA	118
98	chr4:139483009-139483139	YGGYGGTTAGAYGTTTATTTAATTTGTTTG	CTACAAATCCRAAACAACACCTTAAAAACTAAA	131
99	chr8:103629512-103629661	TTTTGTATTTTTTTTAGTAGAGAYGGGTTTTTAT	TTAAAAACCCCTCTCTCTTCCRAATA	150
101	chr8:145106870-145106994	TTTTYGTTTTYGTAGGTATTYGGTTATTTTG	RCCTCCTCACRAAAAAACAACT	125
102	chr8:145106096-145106222	GTGTTTTTTTAAGGTAAGTATAGATGAGGGG	CCCRCAATAAAAATACCTTAAATCTCC	127
103	chr8:145106092-145106211	GATGTTTTAAGTTTTTYGGTTTTGGGGA	CTCAATATCCTCCTAAAACAAACACAAATAAAAAA	120
105	chr8:145103775-145103893	TGTGTAAAGTYGGTGAGGTGTTGA	AAATCCAAAATAAAAATTTAAAATCAAATCCCTTT	119
106	chr9:100069971-100070085	GGTAGAGTGAAGTATAAGTAATAATTTTGTATTATT	AAATAAACTCTAAACTCRAACAAAAAAC	115
107	chr9:100069972-100070073	AATTYGAGTAGGAGGTYGGTTTTTTT	ACAAAATAAAACACAAACAATAATCCTATATTATT	102
108	chr9:139553849-139553943	ATTTTTTTTTTTTGTATTTTTTGTAGTTYGGTTTT	CAAAAACCAACCTAAACATCTCAAATAA	95
109	chr9:139553853-139553951	GTTTTTGGTAGAGGTTAGTTTGGGTATTTTAGGTGG	CCTCCTCCTACACCTCCTACAACC	99
110	chr11:62693570-62693687	TAGTTTTYGAAGAAAAGATGGGTTTGGGG	AACRCCRAATCAACTCAACRAAATCTC	118
111	chr11:62693550-62693659	GGTYGGGTTATAAGGATTYGGGAA	CCAACCCCAAATCTTACRAACAATTCC	110
112	chr12:49390739-49390861	GGAGTYGTTATGGAYGTTGAGTTTTTTAGTTT	TAAACRCRAACCCCAAAACCCTA	123
113	chr12:49390712-49390852	ATTTTAGGGTTTTAYGTYGGGGTTTTG	CRAAACTTCTATATCRCTTCCATCAAAAAAAC	141
114	chr16:1271174-1271302	GTTTTAYGAGTTTTYGTTYGTTTTGGTT	AATACCCRATCTTCRTCCCACAAA	129
115	chr16:1271197-1271314	TTTTTTTTGGYGGGTGTTYGGTTTT	TAATTCRAATTTCTCCRAATTTTACTACCAAC	118
116	chr17:43037200-43037354	TTTTYGTGTTAGGGAATYGGGGTTGAT	CTCATCTCAAAACRCAAAAAACAAACC	155
117	chr17:59532206-59532307	GTTTTTTTTTGYGATTTYGTAGGT	CRCTAAATCCAACTATATCTAAAACCC	102
118	chr17:59532225-59532309	YGYGTTGGGTTTAATTATATTTAGGGTTT	CAAACCRCCACATCTAAAACCAA	85
119	chr19:17439718-17439872	TYGGTATAGGTGYGGTTGTAGGATTT	GCCCAAAAAAAATAAATCCCCCRATCC	155
120	chr19:17439774-17439875	GGYGTTTAGGGAGGGTGAGTTTTT	TCAACAACRTAACCAACATCTAAAAACAAA	102
121	chrX:152245134-152245286	ATTTTTYGGTYGAGTTYGATTAGGGT	CATCCTAAACATCCCRAAAAACTATAATAATAC	153
122	chrX:152245154-152245280	GGGTATTTYGGAGGATTGTGATGATGT	CAAAACCRCRATAACATTATCCTCCTCT	127

Table S1 Coordinates and primers used to amplify the identified target regions using bisulfite sequencing.
